# Polymer Conductive Membrane-Based Non-Touch Mode Circular Capacitive Pressure Sensors: An Analytical Solution-Based Method for Design and Numerical Calibration

**DOI:** 10.3390/polym14153087

**Published:** 2022-07-29

**Authors:** Fei-Yan Li, Qi Zhang, Xue Li, Xiao-Ting He, Jun-Yi Sun

**Affiliations:** 1 School of Civil Engineering, Chongqing University, Chongqing 400045, China; 202116131224t@cqu.edu.cn (F.-Y.L.); 202016021045@cqu.edu.cn (Q.Z.); 20161602025t@cqu.edu.cn (X.L.); hexiaoting@cqu.edu.cn (X.-T.H.); 2 Key Laboratory of New Technology for Construction of Cities in Mountain Area (Chongqing University), Ministry of Education, Chongqing 400045, China

**Keywords:** capacitive pressure sensor, polymer conductive membrane, large deflection, analytical solution, numerical calibration

## Abstract

In this paper, an analytical solution-based method for the design and numerical calibration of polymer conductive membrane-based non-touch mode circular capacitive pressure sensors is presented. The accurate analytical relationship between the capacitance and applied pressure of the sensors is derived by using the analytical solution for the elastic behavior of the circular polymer conductive membranes under pressure. Based on numerical calculations using the accurate analytical relationship and the analytical solution, the analytical relationship between the pressure as output and the capacitance as input, which is necessary to achieve the capacitive pressure sensor mechanism of detecting pressure by measuring capacitance, is accurately established by least-squares data fitting. An example of how to arrive at the design and numerical calibration of a non-touch mode circular capacitive pressure sensor is first given. Then, the influence of changing design parameters such as membrane thickness and Young’s modulus of elasticity on input–output relationships is investigated, thus clarifying the direction of approaching the desired input–output relationships by changing design parameters.

## 1. Introduction

Thin films are widely used in many engineering and technical fields, and most of these have good elastic deformation ability and can exhibit large elastic deflection under lateral loading [1,2,3,4,5,6], which provides the possibility for designing and developing thin film elastic deflection-based devices [7,8,9,10,11,12,13,14]. Among them, capacitive pressure sensors are a good example of physical quantity (pressure) detection by deflection measurement. They have advantages of high performance-to-price ratio, high reliability, stability and sensitivity, low power consumption, no turn-on temperature drift, and lower sensitivity to side stress and other environment effects. In microelectromechanical systems (MEMS), they usually use silicon or silicon carbide thin films [15,16,17], polymer/ceramic thin films [18] or low-temperature co-fired ceramics thin films [19], or graphene-polymer heterostructure thin films [20,21,22,23].

**Figure 1:**
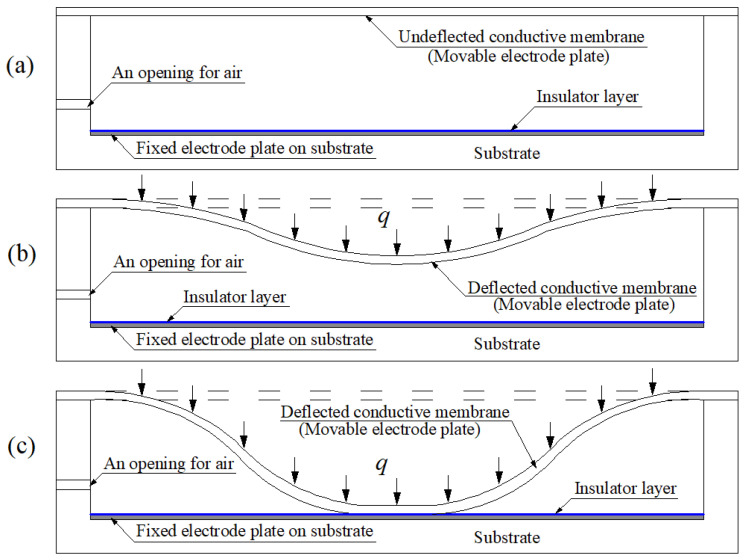
Sketch of the structure and modes of operation of a membrane elastic deflection-based capacitive pressure sensor: (**a**) the initial status without application of the pressure *q*, (**b**) non-touch mode of operation under the pressure *q*, and (**c**) touch mode of operation under the pressure *q*.

The basic structure and modes of operation of a membrane elastic deflection-based capacitive pressure sensor are shown in Figure 1, where the fixed electrode plate on a substrate forms a parallel plate capacitor together with the initially flat undeflected conductive membrane (as a movable electrode plate of the capacitor). On application of pressure *q*, the conductive membrane elastically deflects towards the fixed electrode plate, making the initial parallel plate capacitor become a non-parallel plate capacitor and resulting in a change in capacitance of the capacitor. Before the conductive membrane touches the insulator layer coating on the fixed electrode plate, the capacitive pressure sensor is said to operate in non-touch mode or normal mode and called a non-touch mode or normal mode capacitive pressure sensor [24,25,26,27,28,29], as shown in Figure 1b. Additionally, after the conductive membrane touches the insulator layer, the capacitive pressure sensor is said to operate in touch mode and called a touch mode capacitive pressure sensor [23,30,31,32,33], as shown in Figure 1c. Obviously, the applied pressure *q* can be expected to be determined by measuring the capacitance of the non-parallel plate capacitor, due to their one-to-one correspondence (analytical relationship), which is the basic principle of such capacitive pressure sensors.

However, the analytical relationship between the capacitance of the non-parallel plate capacitor and the applied pressure is very difficult to be exactly established due to the strong nonlinearity of the elastic behavior of the deflected conductive membrane under pressure. So, various approximation methods have to be used to obtain approximate analytical relationships between capacitance and pressure. In particular, the non-parallel plate capacitor with touch mode of operation is often simplified as an equivalent parallel plate capacitor, where only the capacitance in the touched area of the insulator layer and conductive membrane is considered and the capacitance in the untouched area is ignored [23,30,31], because the effective gap between the fixed electrode plate and conductive membrane is the thickness of the insulator layer, and the insulator layer can be designed to be very thin and have a very large dielectric constant. Furthermore, the touched area was also assumed to be approximately proportional to the applied pressure [30]. This makes it possible to establish a nearly linear analytical relationship between capacitance and pressure. On the other hand, because the non-parallel plate capacitor with non-touch mode of operation has an intrinsic nonlinear capacitance–pressure relationship, many efforts have been made to reduce its nonlinear characteristic either by modifying the shape of the fixed electrode plate [25,26,27,34] or by using special nonlinear converter circuits [29,35]. However, the existing studies often suggest that non-touch mode capacitive pressure sensors are far inferior to touch mode capacitive pressure sensors in terms of the easy realization of nearly linear capacitance–pressure relationships [30]. However, it should also be pointed out that the nearly linear capacitance–pressure relationships of the touch mode or non-touch mode capacitive pressure sensors in the literature all apply only to a certain pressure range; that is, these sensors are designed to linearly operate within a certain pressure range, and beyond this pressure range, they are still nonlinear. In other words, their capacitance–pressure relationships are nearly linear in a certain pressure range and, from a point of view beyond this pressure range, are still nonlinear. However, such a segment of nearly linear capacitance–pressure relationships is, in fact, not very difficult to achieve, as long as the analytical solution for the elastic behavior of the circular conductive membrane under pressure can be obtained, which can be seen later in Section 3.

In this study, an analytical solution-based method for design and numerical calibration of polymer conductive membrane-based non-touch mode circular capacitive pressure sensors is presented. The circular polymer conductive membranes are used as the pressure sensing elements, the movable electrode plates, of capacitive pressure sensors. They are usually fixed at their circular peripheries, thus will exhibit axisymmetric deformation with large deflection when subjected to a uniform differential pressure between their upper and lower opposite surfaces. By controlling the range of pressure applied, they do not touch the fixed electrode plate of the sensors so as to keep the non-touch mode of operation. Due to the fact that their upper and lower opposite surfaces are simultaneously stretched during deflection, there is no compressive stress at all but only tensile stress on their cross sections. Therefore, the elastic behavior of free deflection of the circular polymer conductive membranes under pressure can be regarded as a problem of axisymmetric deformation with large deflection of an initially flat, peripherally fixed circular membrane under uniformly distributed transverse loads. Essential to the design and numerical calibration of such non-touch mode circular capacitive pressure sensors is the analytical solutions of stress and deflection for this axisymmetric deformation problem. In this paper, they are accurately derived, and the obtained analytical solution of stress is used to determine the maximum pressure allowed to be applied to the non-touch mode circular capacitive pressure sensors, which depends on the yield strength of the circular membranes. The accurate analytical relationship between the total capacitance and applied pressure of the sensors is derived by using the analytical solution of deflection and is given in the form of the integral of the membrane deflection that is a strongly nonlinear function of the applied pressure. Therefore, in order to achieve the capacitive pressure sensor mechanism of detecting pressure by measuring capacitance, the accurate analytical relationship between the pressure as output and the capacitance as input is given by using the least-squares data fitting based on numerical calculations.

The analytical solution-based method presented here can make the non-touch mode circular capacitive pressure sensors be more accurately designed and numerically calibrated, thus greatly reducing the dependence on experimental calibrations. In comparison with the methods in the literature such as modifying the shape of substrate electrode plates [25,26,27,34] or using special nonlinear converter circuits [29,35], this novel method has the advantages of intuition, clarity, strong tunability and operability. By changing design parameters, including geometric parameters (such as the thickness of the circular membranes and the initial gap between initially flat undeflected circular membranes and fixed electrode plates) and physical parameters (such as the Poisson’s ratio and Young’s modulus of elasticity of the circular membranes), it can easily realize the accurate analytical relationships between the pressure as output and the capacitance as input, including linear and non-linear relationships. Therefore, from this point of view, the view in the literature is open to debate that non-touch mode capacitive pressure sensors are far inferior to touch mode capacitive pressure sensors in the easy realization of nearly linear input–output relationships [30]. This should be due to the lack of the exact analytical solutions and their effective applications.

The paper is organized as follows. In the following section, the accurate analytical relationship between the total capacitance and applied pressure of the non-touch mode circular capacitive pressure sensors is derived in detail, the analytical solutions of stress and deflection for the elastic behavior of free deflection of the circular conductive membranes under pressure are accurately derived, and how to design and numerically calibrate the non-touch mode circular capacitive pressure sensors is described in detail. In Section 3, an example is first given of how to arrive at a design and numerical calibration of non-touch mode circular capacitive pressure sensors. Then, in order to clarify the direction of approaching the desired pressure–capacitance relationships by changing design parameters, the influence of changing design parameters on pressure–capacitance relationships is investigated. Concluding remarks are given in Section 4.

## 2. Materials and Methods

**Figure 2:**
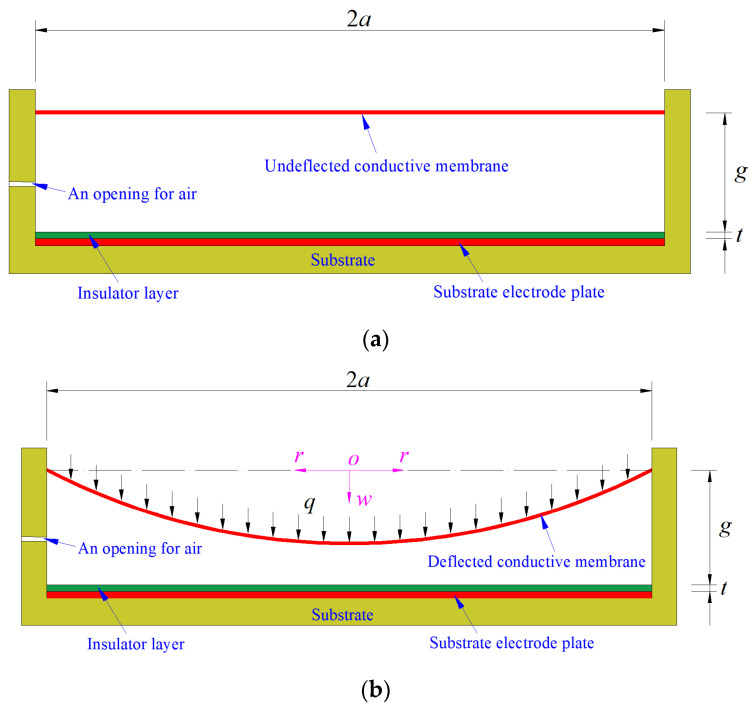
Sketch of the capacitive pressure sensor: (**a**) initial state and (**b**) operating state.

The geometry and configuration of a non-touch mode circular capacitive pressure sensor is shown in Figure 2a, where the initially flat, undeflected, circular conductive membrane with Poisson’s ratio *v*, Young’s modulus of elasticity *E*, thickness *h* and radius *a* forms a parallel plate capacitor together with the electrode plate fixed to the substrate, *t* denotes the thickness of the insulator layer coating on the substrate electrode plate, and *g* denotes the initial gap between the insulator layer and the initially flat, undeflected, circular conductive membrane. On application of pressure (the uniformly distributed transverse loads *q*), as shown in Figure 2b, the initially flat, undeflected, circular conductive membrane deflects towards the substrate electrode plate, making the initial parallel plate capacitor become a non-parallel plate capacitor and resulting in a change in capacitance of the capacitor. In Figure 2b, the dash-dotted line represents the plane in which the geometric middle plane of the initially flat, undeflected, circular conductive membrane is located, *o* denotes the origin of the introduced cylindrical coordinate system (*r*, *φ*, *w*), *r* is the radial coordinate, *φ* is the angle coordinate but not represented in Figure 2b, and *w* is the axial coordinate and denotes the deflection of the deflected conductive membrane.

Before the pressure *q* is applied to the circular conductive membrane, the total initial capacitance *C*_0_ of the initial parallel plate capacitor formed by the initially flat, undeflected, circular conductive membrane and the substrate electrode plate comprises the capacitance *C*_1_ and *C*_2_ of two series parallel plate capacitors, where *C*_1_ refers to the capacitance of the parallel plate capacitor with the insulator layer gap *t* and relative permittivity *ε_r_*_1_, and *C*_2_ refers to the capacitance of the parallel plate capacitor with the air gap *g* and relative permittivity *ε_r_*_2_. Therefore, if the vacuum permittivity is denoted by *ε*_0_, then

(1) $$\frac{1}{C_{0}}=\frac{1}{C_{1}}+\frac{1}{C_{2}},$$

where

(2) $$C_{1}=\frac{\varepsilon_{0}\varepsilon_{r1}\pi a^{2}}{t}$$

and

(3) $$C_{2}=\frac{\varepsilon_{0}\varepsilon_{r2}\pi a^{2}}{g}.$$

Thus,

(4) $$C_{0}=\frac{C_{1}C_{2}}{C_{1}+C_{2}}=\frac{\frac{\varepsilon_{0}\varepsilon_{r2}\pi a^{2}}{t}\frac{\varepsilon_{0}\varepsilon_{r1}\pi a^{2}}{g}}{\frac{\varepsilon_{0}\varepsilon_{r2}\pi a^{2}}{t}+\frac{\varepsilon_{0}\varepsilon_{r1}\pi a^{2}}{g}}=\frac{\varepsilon_{0}\varepsilon_{r1}\varepsilon_{r2}\pi a^{2}}{\varepsilon_{r1}t+\varepsilon_{r2}g}.$$

**Figure 3:**
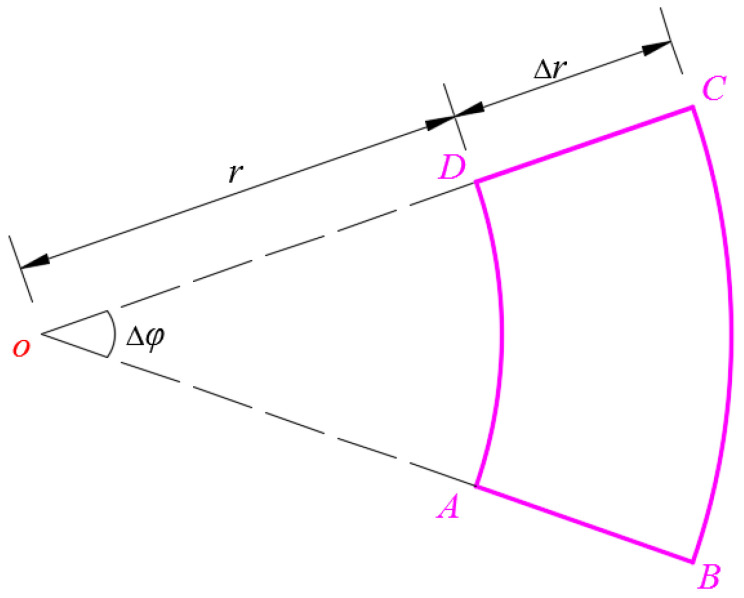
Sketch of the micro area element ABCD taken from the substrate electrode plate.

After the pressure *q* is applied to the conductive membrane, the total capacitance *C* of the non-parallel plate capacitor formed by the deflected circular conductive membrane and the substrate electrode plate is still composed of the capacitance of two series capacitors: one is the capacitance *C*_1_ of the parallel plate capacitor with the insulator layer gap *t* and relative permittivity *ε_r_*_1_, which is still given by Equation (2); the other is the capacitance ${C^{\prime }}_{2}$ of the air dielectric non-parallel plate capacitor with the relative permittivity *ε_r_*_2_ and uneven distribution of air gap *g*–*w*(*r*) (see Figure 2b). Therefore, the expression of capacitance ${C^{\prime }}_{2}$ needs to be further derived. To this end, let us take a micro area element, ABCD, from the substrate electrode plate, as shown in Figure 3.

The area of the micro area element ABCD is

(5) $$\Delta S=\frac{{\left(r+\Delta r\right)}^{2}\Delta \varphi }{2}-\frac{r^{2}\Delta \varphi }{2}=r\Delta r\Delta \varphi +\frac{1}{2}{\left(\Delta r\right)}^{2}\Delta \varphi .$$

After ignoring the higher-order terms (the second term in Equation (5)), Δ*S* can be approximated by *r*Δ*r*∆*φ*, while the air gap between this micro area element ABCD on the substrate electrode plate and the corresponding deflected conductive membrane can be approximated by *g* − *w*(*r*), resulting in

(6) $$\Delta {C^{\prime }}_{2}=\varepsilon_{0}\varepsilon_{r2}\frac{r\Delta r\Delta \varphi }{g-w(r)}$$

and

(7) $${C^{\prime }}_{2}=\int_{0}^{a}\int_{0}^{2\pi }\varepsilon_{0}\varepsilon_{r2}\frac{r}{g-w(r)}d\varphi dr=2\pi \varepsilon_{0}\varepsilon_{r2}\int_{0}^{a}\frac{r}{g-w(r)}dr.$$

Thus, the total capacitance *C* of the non-parallel plate capacitor formed by the deflected circular conductive membrane and the substrate electrode plate may finally be written as

(8) $$C=\frac{C_{1}{C^{\prime }}_{2}}{C_{1}+{C^{\prime }}_{2}}=\frac{\frac{\varepsilon_{0}\varepsilon_{r1}\pi a^{2}}{t}2\pi \varepsilon_{0}\varepsilon_{r2}\int_{0}^{a}\frac{r}{g-w(r)}dr}{\frac{\varepsilon_{0}\varepsilon_{r1}\pi a^{2}}{t}+2\pi \varepsilon_{0}\varepsilon_{r2}\int_{0}^{a}\frac{r}{g-w(r)}dr}.$$

It can be seen from Equation (8) that the total capacitance *C* can be determined as long as an analytical expression for deflection *w*(*r*) is available. Therefore, the analytical solutions of deflection *w*(*r*) and stress *σ_r_*(*r*) of the deflected circular conductive membrane under pressure *q* is vital to the determination of the total capacitance *C* of the non-parallel plate capacitor formed by the deflected circular conductive membrane under pressure *q* and the substrate electrode plate. 

To this end, we have to analytically solve the problem of axisymmetric deformation with large deflection of the deflected circular conductive membrane under the uniformly distributed transverse loads *q*. However, for the sake of coherence, the detailed derivation of the analytical solution of this axisymmetric deformation problem is arranged in the Appendix A. The analytical expressions for stress *σ_r_*(*r*) and deflection *w*(*r*) can be written as, from Equations (A16), (A22) and (A23),

(9) $$\sigma_{r}(r)=E\sum_{i=0}^{\infty }\frac{b_{2i}}{a^{2i}}r^{2i}$$

and

(10) $$w(r)=\sum_{i=0}^{\infty }\frac{c_{2i}}{a^{2i-1}}r^{2i},$$

where *c_*2*i_* and *b_*2*i_* are the coefficients of the power series, which are listed in Appendix B. It can be seen from Appendix B that when *i* ≠ 0 the coefficients *c_*2*i_* and *b_*2*i_* are expressed into the polynomials with regard to the coefficients *b*_0_, Poisson’s ratio *v* and dimensionless parameter *Q* (the dimensionless pressure, see Equation (A16)). The coefficients *b*_0_ and *c*_0_ are usually called undetermined constants. For a given Poisson’s ratio *v*, Young’s modulus of elasticity *E*, thickness *h*, radius *a* and pressure *q*, the undetermined constant *b*_0_ can be determined by solving Equation (A24). Additionally, with the known *b*_0_, all the coefficients *c_*2*i_* and *b_*2*i_* when *i* ≠ 0 can be determined (see Appendix B), such that the undetermined constant *c*_0_ can be determined by Equation (A25). In this way, the deflection expression, i.e., Equation (10), can be determined due to the known coefficient *c_*2*i_* (*i* = 0, 1, 2, 3…). The maximum stress *σ*_m_ and maximum deflection *w*_m_ of the axisymmetrically deflected circular conductive membrane are at its center (i.e., at *r* = 0), hence given by

(11) $$\sigma_{m}=Eb_{0}$$

and

(12) $$w_{m}=ac_{0}.$$

For a given conductive membrane (given Poisson’s ratio *v*, Young’s modulus of elasticity *E*, thickness *h*, radius *a* and yield strength *σ*_y_), the maximum stress *σ*_m_ at any pressure *q* can be determined by Equation (11). To ensure the strength of the material, it is assumed that the working stress of the conductive membrane is always controlled below 70% of the yield strength *σ*_y_. So, if the pressure *q* at *σ*_m_ = 0.7*σ*_y_ is equal to the maximum pressure of a given pressure measurement range, then the given conductive membrane meets the design requirements; otherwise, a new conductive membrane (with different design parameters such as membrane thickness *h*, Poisson’s ratio *v* and Young’s modulus of elasticity *E*) needs to be selected. On the other hand, the maximum deflection *w*_m_ at *σ*_m_ = 0.7*σ*_y_ can be determined by Equation (12) and is used primarily to determine the initial gap *g* between the insulator layer and the initially flat, undeflected, circular conductive membrane, see Figure 2a. The minimum value of the initial gap *g* should be greater than but as close as possible to this maximum deflection *w*_m_.

After plugging the known deflection expression (i.e., for given Poisson’s ratio *v*, Young’s modulus of elasticity *E*, thickness *h*, radius *a* and pressure *q*, the power series coefficients *c*_2*i*_/*a*^2*i*−1^ in Equation (10) are known) into Equation (8), the total capacitance *C* of the non-parallel plate capacitor, which is formed by the deflected circular conductive membrane under the given pressure *q* and the substrate electrode plate, can finally be determined with the known initial gap *g*, vacuum permittivity *ε*_0_, and relative permittivities *ε_r_*_1_ and *ε_r_*_2_. In this way, a pair of numerical values of calculated capacitance *C* and given loads *q*, having an intrinsic analytical relationship, is thus established. Additionally, with another given value of pressure *q*, another pair of numerical values of calculated capacitance *C* and given loads *q* can be further established. 

Therefore, the numerical calculations of a progressive increase in the values of pressure *q* will result in a data sequence (sequential number pairs) with respect to numerical values of calculated capacitance *C* and given loads *q*, as shown in the next section. Additionally, further, based on this data sequence, the analytical relationship between loads *q* and capacitance *C* can be established by using least-squares data fitting, including straight line fitting and curve fitting, as shown in the next section. In each fitting function, the ranges of variation of loads *q* and capacitance *C* are affected by different requirements of fitting accuracy (average sum of fitting error squares). On the other hand, for given requirements of fitting accuracy, the ranges of variation of loads *q* and capacitance *C* can also be changed by changing geometric parameters (such as the thickness *h* and radius *a* of the conductive membranes and the initial gap *g*) and physical parameters (such as the Poisson’s ratio *v* and Young’s modulus of elasticity *E* of the conductive membranes), as shown in Section 3.2. 

All in all, with Equation (8) and the analytical solution in Appendix A, the non-touch mode circular capacitive pressure sensors can be perfectly designed and numerically calibrated, thus greatly reducing the dependence on experimental calibration.

## 3. Results and Discussion

In this section, an example is first given of how to use Equation (8) and the analytical solution in Appendix A to realize the design and numerical calibration of non-touch mode circular capacitive pressure sensors (see Section 3.1). Then, in order to clarify the direction of approaching the desired pressure–capacitance relationships by changing design parameters, the influence of changing design parameters on pressure–capacitance relationships is comprehensively investigated, such as changing the initial gap *g* between the insulator layer coating on the substrate electrode plate and the initially flat undeflected circular conductive membrane, the thickness *h* of the circular conductive membranes, Young’s modulus of elasticity *E*, Poisson’s ratio *v* and the thickness *t* of the insulator layers, see Section 3.2. 

In fact, Equation (8) has given the accurate analytical relationship between the capacitance *C* and the pressure *q*, where *q* is included in the power series coefficients *c_*2*i_* of the deflection *w*(*r*) (see Appendix B). However, in order to achieve the sensor mechanism of detecting pressure by measuring capacitance, we need to know the accurate analytical relationship between the pressure *q* as output and the capacitance *C* as input, that is, the analytical expression of the capacitance *C* as independent variable and the pressure *q* as dependent variable, *q*(*C*). Obviously, such an analytical expression cannot be directly given due to the strong nonlinearity between the deflection *w*(*r*) and the applied pressure *q*. Therefore, in this case, we have to perform a lot of numerical calculations using Equation (8) and the analytical solution of deflection and use least-squares data fitting to arrive at the analytical expression *q*(*C*), which may be seen in Section 3.1.

On the other hand, the numerical calculations using Equation (8) and the analytical solution of deflection can only be carried out on the premise that the circular conductive membrane is known and the range of pressure *q* is specified. Therefore, the design of a non-touch mode circular capacitive pressure sensor whose pressure range is beforehand specified has to begin with a tentative choice of a circular conductive membrane, including membrane thickness *h*, Poisson’s ratio *v* and Young’s modulus of elasticity *E*. If the resulting pressure–capacitance relationship, *q*(*C*), does not satisfy the desired usage or technical requirements, especially the range of the input capacitance *C* and output pressure *q*, then the design parameters, especially the membrane thickness *h* and Young’s modulus of elasticity *E*, must be adjusted. Section 3.2 gives the direction of the adjustment for approaching the desired usage or technical requirements.

### 3.1. An Example of Design and Numerical Calibration Based on Analytical Solutions

**Figure 4:**
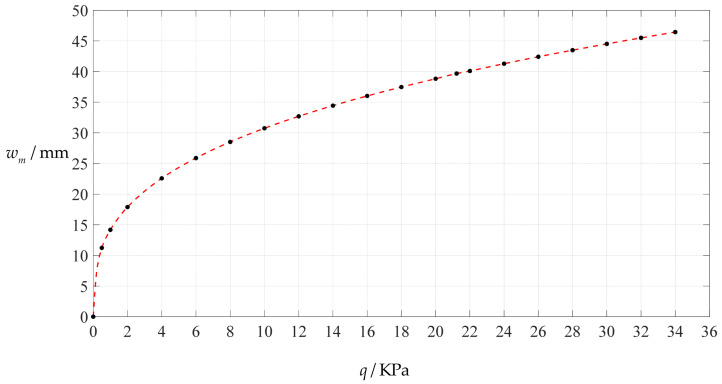
Variation of maximum deflection *w_m_* with pressure *q*.

**Figure 5:**
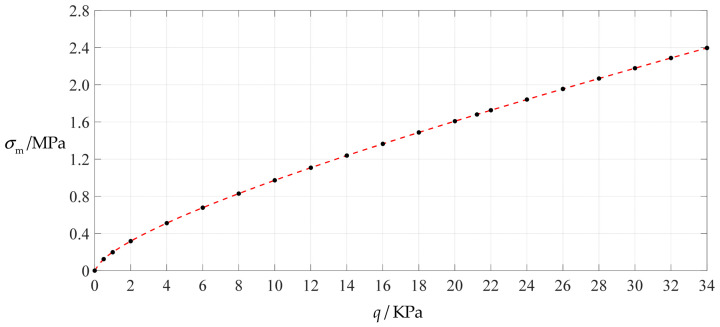
Variation of maximum stress *σ*_m_ with pressure *q*.

A non-touch mode circular capacitive pressure sensor is assumed to use a circular conductive membrane with Poisson’s ratio *v* = 0.47, Young’s modulus of elasticity *E* = 7.84 MPa, radius *a* = 100 mm, thickness *h* = 1 mm and yield strength *σ*_y_ = 2.4 MPa. The maximum value of the applied pressure *q* can be determined by the condition that the maximum stress *σ*_m_ of the circular conductive membrane under pressure *q* does not exceed its yield strength *σ*_y_ = 2.4 MPa. Table 1 shows the calculation results as the applied pressure *q* progressively increases, where the undetermined constants *b*_0_ and *c*_0_ are calculated by Equations (A24) and (A25), the maximum stress *σ*_m_ and maximum deflection *w*_m_ are calculated by Equations (11) and (12). It may be seen from Table 1 that when the maximum stress *σ*_m_ approaches the yield strength *σ*_y_ = 2.4 MPa, the maximum value of the applied pressure *q* is about 34 KPa. Figure 4 and Figure 5 show the variations of *w*_m_ and *σ*_m_ with the applied pressure *q*. 

If the working stress of the circular conductive membrane is always controlled to be less than or equal to 70% of the yield strength *σ*_y_, that is, *σ*_m_ ≤ 0.7 *σ*_y_ ≈ 1.68 MPa, then it can be seen from Table 1 that the maximum operation pressure should not exceed 21.225 KPa. Therefore, the values of the undetermined constants *b*_0_ at pressures less than or equal to 21.225 KPa in Table 1 will be used to determine the values of the coefficients *c_*2*i_* (see Appendix B for their expressions), as shown in Table 2 and Table 3. Moreover, from Table 1, we may also see that the value of the maximum deflection *w*_m_ corresponding to 21.225 KPa pressure is about 39.67 mm. Therefore, the initial gap *g* between the initially flat undeflected conductive membrane and the insulator layer coating on the substrate electrode plate should be greater than or equal to 41 mm. For investigating the influence of changing the initial gap *g* on the input–output relationship between the input capacitance *C* and the output pressure *q*, the pressure–capacitance relationship *q*(*C*), here, the initial gap *g* takes 41 mm, 46 mm and 51 mm, respectively.

If the insulator layer is assumed to take 0.1 mm of polystyrene, then *t* = 0.1 mm and the relative permittivity *ε*_r1_ = 2.5. In addition, the vacuum permittivity *ε*_0_ = 8.854 × 10^−12^ F/m = 8.854 × 10^−3^ pF/mm, and the air relative permittivity *ε*_r2_ = 1.00053. The deflection expressions describing the shape of the deflected conductive membrane under different pressures *q* can be determined by Equation (10) with the values of the coefficients *c_*2*i_* in Table 2 and Table 3. Therefore, the values of the total capacitance (at rest) of the non-parallel plate capacitor formed by the deflected circular conductive membrane and the substrate electrode plate may finally be determined by Equation (8), which are listed in Table 4, where the definite integral in Equation (8) was calculated by using Maple 2018 software package.

**Figure 6:**
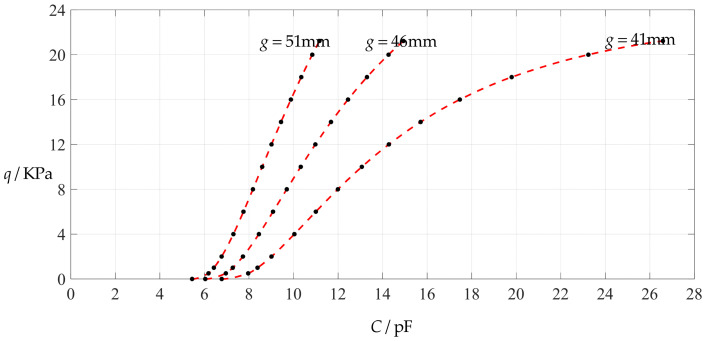
Variations of pressure *q* with capacitance *C*, when *a* = 100 mm, *h* = 1 mm, *E* = 7.84 MPa, *ν* = 0.47, *t* = 0.1 mm, and *g* = 41 mm, 45 mm and 51 mm.

**Figure 7:**
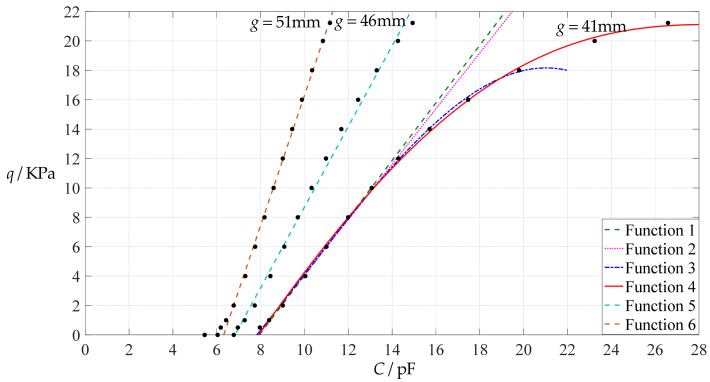
Least-squares fitting of the relationships between *q* and *C* in Figure 6.

Figure 6 shows the variations of pressure *q* with capacitance *C*, showing that the increase in the initial gap *g* will increase the degree of linearity of the pressure–capacitance relationship *q*(*C*). From this point of view, the view in the literature is open to debate that non-touch mode capacitive pressure sensors are far inferior to touch mode capacitive pressure sensors in the easy realization of nearly linear input–output relationships [30]. The linearization in such a way, however, will narrow the range of the input capacitance and eventually increase the output pressure per unit capacitance, in addition to increasing the edge effect in capacitance of the non-parallel plate capacitor. So, it is best not to do so unless necessary. In fact, it can be imagined from Figure 6 that the nearly linear pressure–capacitance relationship *q*(*C*) can also be realized by least-squares data fitting of the data for *g* = 41 mm. Figure 7 shows the results of least-squares fitting, where Functions 1–4 are the results for *g* = 41 mm, Function 5 is the result for *g* = 46 mm, Function 6 is the result for *g* = 51 mm and Functions 1, 5 and 6 are fitted by straight lines, and Function 2 is fitted by a quadratic function, Function 3 by a cubic function and Function 4 by a quartic function. The resulting fitting functions are listed in Table 5, and the average sum of fitting error squares of each fitting function is shown in the footer of Table 5.

As can be seen from Table 5 and Figure 7, the above design and numerical calibration can realize five non-touch mode circular capacitive pressure sensors with different pressure–capacitance relationships, two linear (Functions 1 and 6) and three nonlinear (Functions 2–4). Obviously, Function 1 should be preferred to Function 6 if a 1~8 KPa pressure range is sufficient for use, because the output pressure per unit capacitance is about 1.940 KPa/pF for Function 1 but 4.267 KPa/pF for Function 6 (which are calculated from Table 5). However, for today’s advanced digital technologies, the emphasis on nearly linear input–output relationships makes no sense, because in most cases, using digital technologies is feasible. Therefore, in this sense, Function 4 should be one of the best choices for pressure monitoring microcomputer systems based on such non-touch mode circular capacitive pressure sensing devices.

Of course, Functions 1–4 and 6 may also not satisfy the usage or technical requirements of the input capacitance and output pressure under consideration. In this case, the design parameters, other than the initial gap *g*, should further be adjusted to meet the desired requirements, as shown in the next section.

### 3.2. Parametric Analysis

As mentioned above, although the increase in the initial gap *g* between the initially flat undeflected conductive membrane and the substrate electrode plate can increase the degree of linearity of the analytical relationship between input capacitance *C* and output pressure *q*, it is not a preferred option to encourage adoption. On the other hand, however, we should also see that decreasing the initial gap *g* can increase the range of input capacitance *C*, see Figure 6. The main purpose of this section is to show the influence of changing the design parameters other than the initial gap *g* on the analytical relationship between input capacitance *C* and output pressure *q*. To this end, we take the design parameters used in Section 3.1 as reference and change each parameter one by one on this basis, such as changing the thickness *h* of the conductive membranes, Young’s modulus of elasticity *E*, Poisson’s ratio *v*, and the thickness *t* of insulator layers.

#### 3.2.1. Effect of Membrane Thickness on Input–Output Relationships

**Figure 8:**
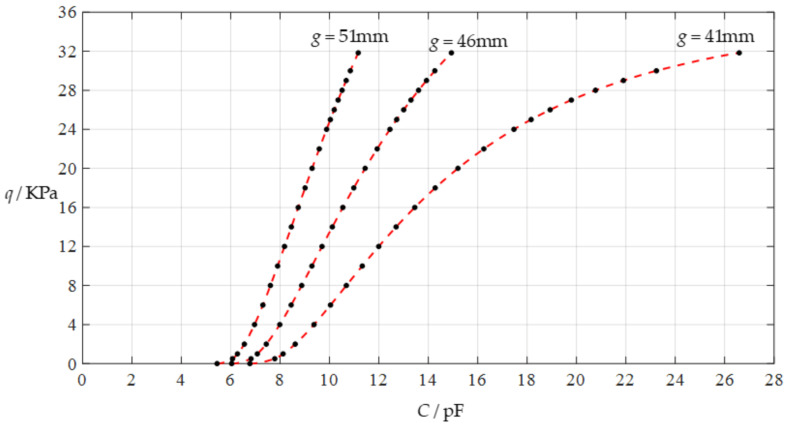
Variations of pressure *q* with capacitance *C*, when *a* = 100 mm, *h* = 1.5 mm, *E* = 7.84 MPa, *ν* = 0.47, *t* = 0.1 mm, and *g* = 41 mm, 46 mm and 51 mm.

**Figure 9:**
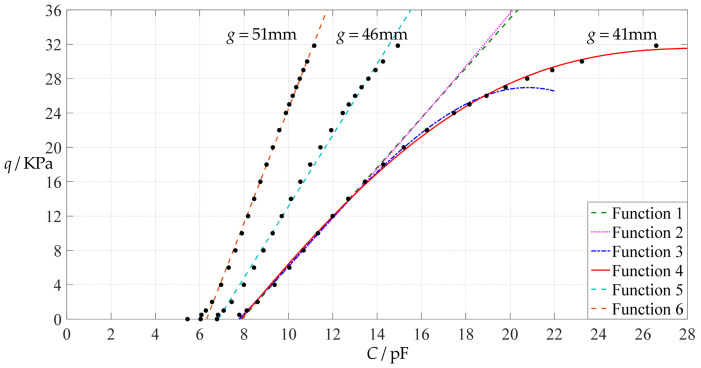
Least-squares fitting of the relationships between *q* and *C* in Figure 8.

**Figure 10:**
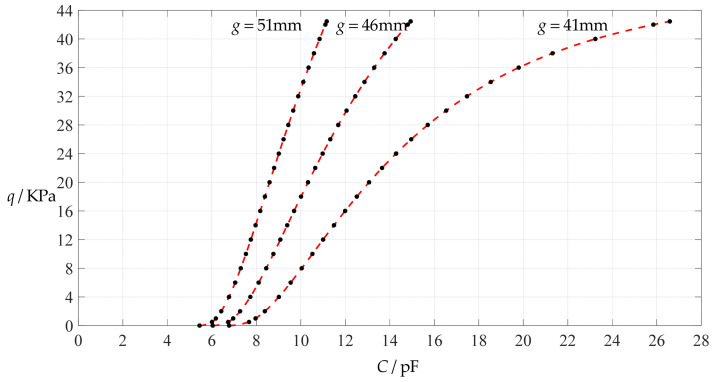
Variations of pressure *q* with capacitance *C*, when *a* = 100 mm, *h* = 2 mm, *E* = 7.84 MPa, *ν* = 0.47, *t* = 0.1 mm, and *g* = 41 mm, 46 mm and 51 mm.

**Figure 11:**
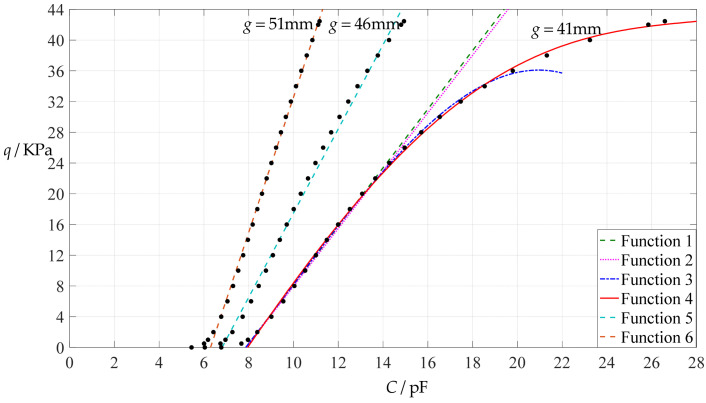
Least-squares fitting of the relationships between *q* and *C* in Figure 10.

**Figure 12:**
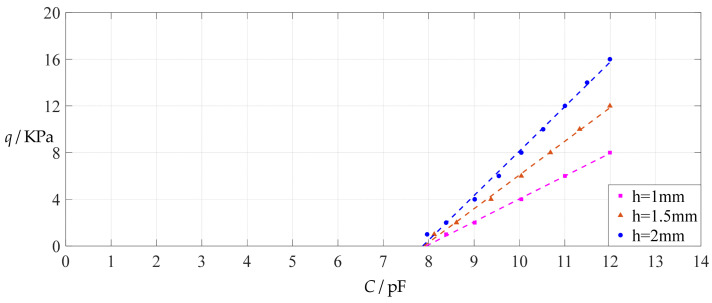
The effect of changing the membrane thickness *h* on Function 1 in Table 5, Table 7 and Table 9 (fitted by a straight line).

**Figure 13:**
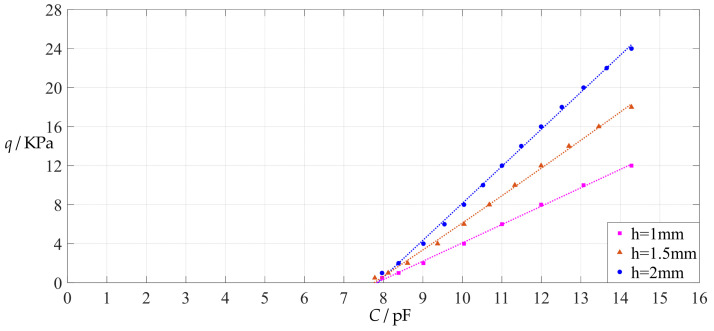
The effect of changing the membrane thickness *h* on Function 2 in Table 5, Table 7 and Table 9 (fitted by a quadratic function).

**Figure 14:**
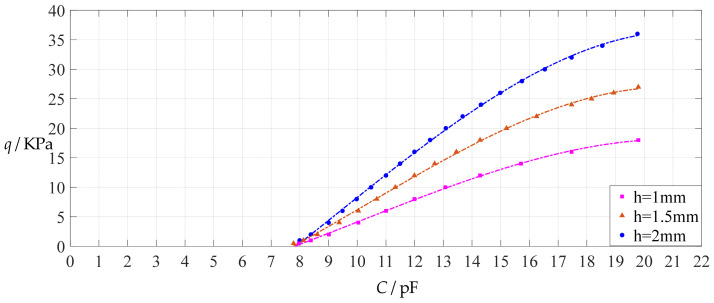
The effect of changing the membrane thickness *h* on Function 3 in Table 5, Table 7 and Table 9 (fitted by a cubic function).

**Figure 15:**
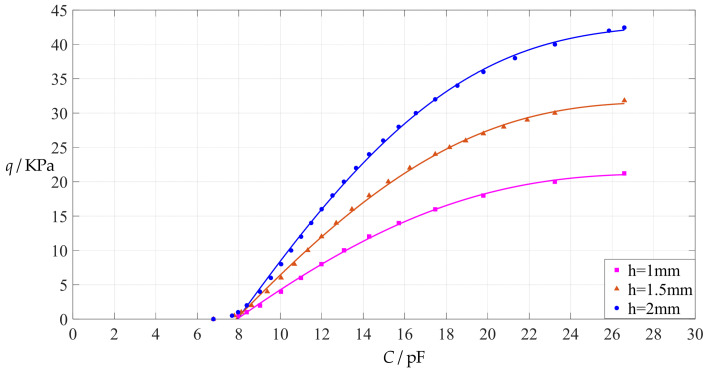
The effect of changing the membrane thickness *h* on Function 4 in Table 5, Table 7 and Table 9 (fitted by a quartic function).

The design parameters used in Section 3.1 are used as reference, that is, Poisson’s ratio *v* = 0.47, Young’s modulus of elasticity *E* = 7.84 MPa, circular conductive membrane radius *a* = 100 mm, circular conductive membrane thickness *h* = 1 mm, insulator layer thickness *t* = 0.1 mm, vacuum permittivity *ε*_0_ = 8.854 × 10^−12^ F/m = 8.854 × 10^−3^ pF/mm, air relative permittivity *ε*_r2_ = 1.00053, insulator layer relative permittivity *ε*_r1_ = 2.5, membrane yield stress *σ*_y_ = 2.4 MPa and membrane maximum stress *σ*_m_ ≤ 0.7 *σ*_y_ ≈ 1.68 MPa. In this section, the thickness *h* of the circular conductive membrane is first increased from the reference thickness of 1 mm to 1.5 mm and then is further increased to 2 mm. When *h* = 1.5 mm, the calculation results are listed in Table 6, the relationships between input capacitance *C* and output pressure *q* are shown in Figure 8, the results of least-squares fitting are shown in Figure 9, the fitting functions are listed in Table 7, and the average sum of fitting error squares of each fitting function is shown in the footer of Table 7. When *h* = 2 mm, the calculation results are listed in Table 8, the input–output relationships are shown in Figure 10, the results of least-squares fitting are shown in Figure 11, the fitting functions are listed in Table 9, and the average sum of fitting error squares of each fitting function is shown in the footer of Table 9. The effects of an increase in the membrane thickness *h* from 1 mm to 1.5 mm and then to 2 mm on the fitting functions (Functions 1–4) are summarized in Figure 12, Figure 13, Figure 14 and Figure 15.

It can be seen from Figure 12, Figure 13, Figure 14 and Figure 15 that the change in the membrane thickness *h* only affects the range of output pressure *q* (increasing with the increase in the membrane thickness *h*) and does not affect the range of input capacitance *C* on the premise of ensuring the basically same fitting accuracy (the average sum of fitting error squares of each fitting function (e.g., Function 1, 2, 3 or 4) is basically the same (see the footers of Table 5, Table 7 and Table 9)). It should also be noted, however, that an increase in the membrane thickness *h* increases the range of output pressure *q*, but it also moderately increases the output pressure per unit capacitance because the input capacitance *C* remains constant. For instance, as the membrane thickness *h* increases from the reference value of 1 mm to 1.5 mm and then to 2 mm, the output pressure per unit capacitance of Function 1 increases from 1.940 KPa/pF to 2.840 KPa/pF and then to 3.724 KPa/pF, while the output pressure per unit capacitance of Function 4 increases from 1.071 KPa/pF to 1.607 KPa/pF and then to 2.143 KPa/pF, which are calculated from Table 5, Table 7 and Table 9.

#### 3.2.2. Effect of Young’s Modulus of Elasticity on Input–Output Relationships 

**Figure 16:**
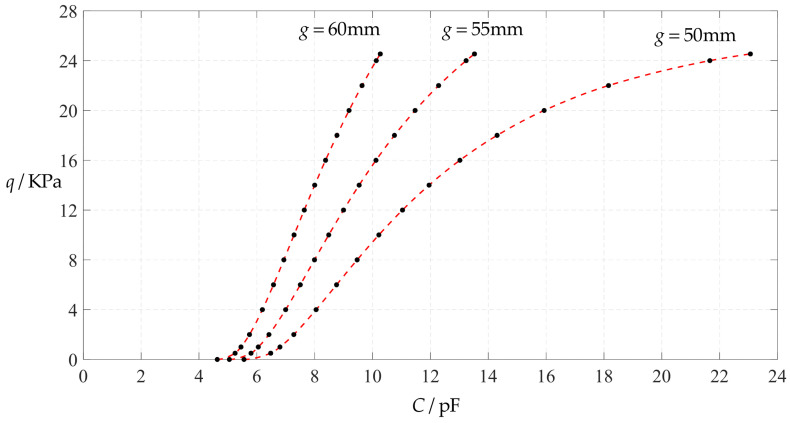
Variations of pressure *q* with capacitance *C*, when *a* = 100 mm, *h* = 1 mm, *E* = 5 MPa, *ν* = 0.47, *t* = 0.1 mm and *g* = 41 mm, 46 mm and 51 mm.

**Figure 17:**
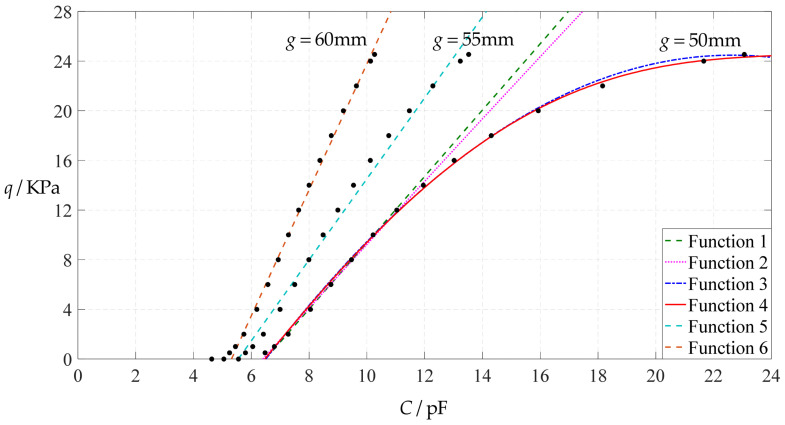
Least-squares fitting of the relationships between *q* and *C* in Figure 16.

**Figure 18:**
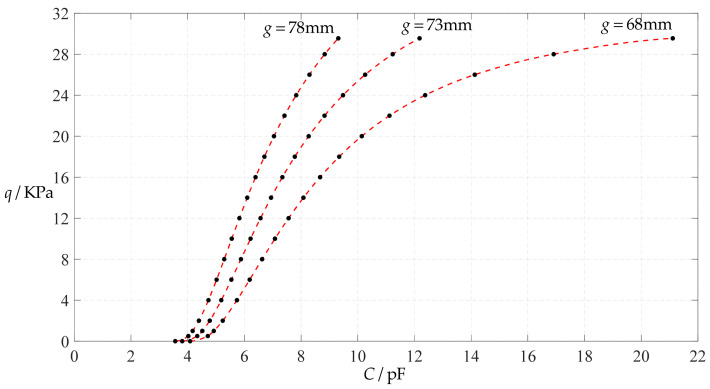
Variations of pressure *q* with capacitance *C*, when *a* = 100 mm, *h* = 1 mm, *E* = 2.5 MPa, *ν* = 0.47, *t* = 0.1 mm, and *g* = 68 mm, 73 mm and 78 mm.

**Figure 19:**
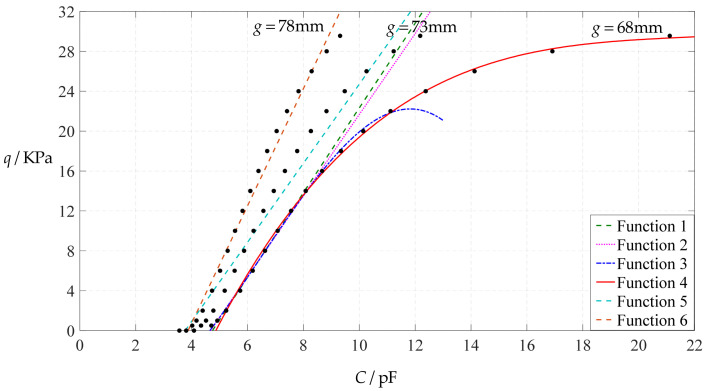
Least-squares fitting of the relationships between *q* and *C* in Figure 18.

**Figure 20:**
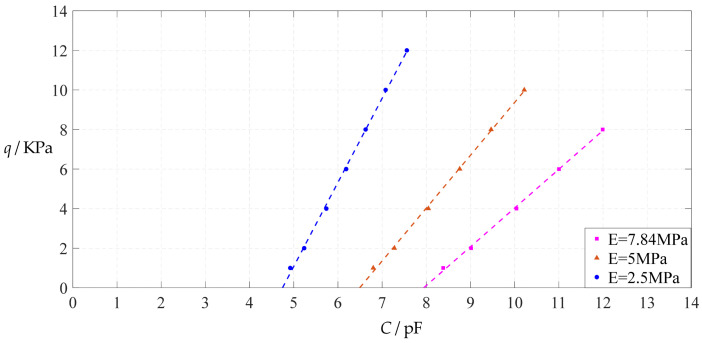
The effect of changing the Young’s modulus of elasticity *E* on Function 1 in Table 5, Table 11 and Table 13 (fitted by a straight line).

**Figure 21:**
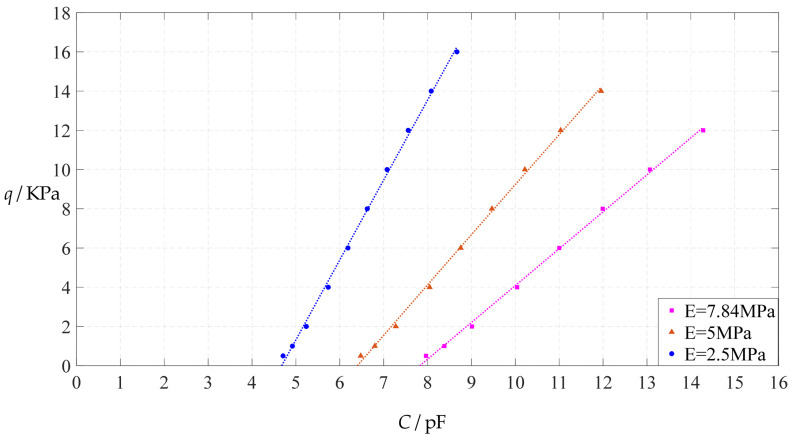
The effect of changing the Young’s modulus of elasticity *E* on Function 2 in Table 5, Table 11 and Table 13 (fitted by a quadratic function).

**Figure 22:**
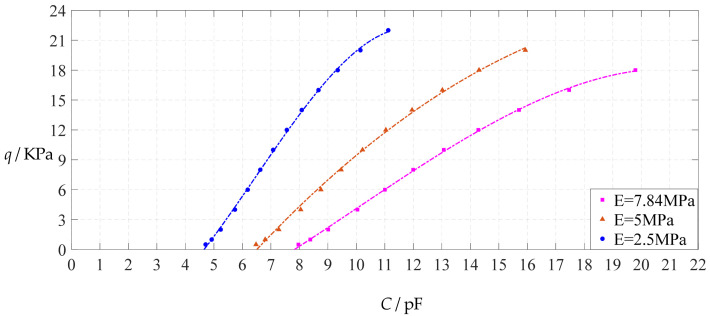
The effect of changing the Young’s modulus of elasticity *E* on Function 3 in Table 5, Table 11 and Table 13 (fitted by a cubic function).

**Figure 23:**
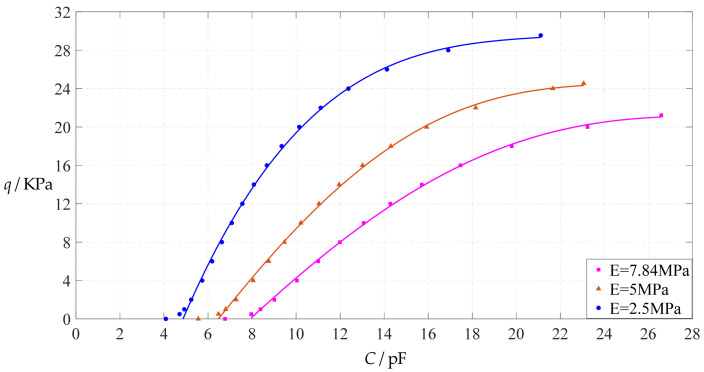
The effect of changing the Young’s modulus of elasticity *E* on Function 4 in Table 5, Table 11 and Table 13 (fitted by a quartic function).

The design parameters used in Section 3.1 are still used as reference, that is, *v* = 0.47, *E* = 7.84 MPa, *a* = 100 mm, *h* = 1 mm, *t* = 0.1 mm, *ε*_0_ = 8.854 × 10^−12^ F/m = 8.854 × 10^−3^ pF/mm, *ε*_r1_ = 2.5, *ε*_r2_ = 1.00053, *σ*_y_ = 2.4 MPa and *σ*_m_ ≤ 0.7 *σ*_y_ ≈ 1.68 MPa. In this section, the Young’s modulus of elasticity *E* of the conductive membrane is first decreased from the reference value of 7.84 MPa to 5 MPa and then further decreased to 2.5 MPa. When *E* = 5 MPa, the calculation results are listed in Table 10, the relationships between input capacitance *C* and output pressure *q* are shown in Figure 16, the results of least-squares fitting are shown in Figure 17, the fitting functions are listed in Table 11, and the average sum of fitting error squares of each fitting function is shown in the footer of Table 11. When *E* = 2.5 MPa, the calculation results are listed in Table 12, the input–output relationships are shown in Figure 18, the results of least-squares fitting are shown in Figure 19, the fitting functions are listed in Table 13, and the average sum of fitting error squares of each fitting function is shown in the footer of Table 13. The effects of a decrease in the Young’s modulus of elasticity *E* from 7.84 MPa to 5 MPa and then to 2.5 MPa on the fitting functions (Functions 1–4) are summarized in Figure 20, Figure 21, Figure 22 and Figure 23.

From Figure 20, Figure 21, Figure 22 and Figure 23, it can be seen that the change in the Young’s modulus of elasticity *E* affects both the range of output pressure *q* (increasing with the decrease in the Young’s modulus of elasticity *E*) and the range of input capacitance *C* (decreasing with the decrease in the Young’s modulus of elasticity *E*) on the premise of ensuring the basically same fitting accuracy (the average sum of fitting error squares of each fitting function (e.g., Function 1, 2, 3 or 4) is basically the same (see the footers of Table 5, Table 11 and Table 13)). Therefore, as the Young’s modulus of elasticity *E* decreases from the reference value of 7.84 MPa to 5 MPa and then to 2.5 MPa, the output pressure per unit capacitance of Function 1 increases from 1.940 KPa/pF to 2.633 KPa/pF and then to 4.168 KPa/pF, while the output pressure per unit capacitance of Function 4 increases from 1.071 KPa/pF to 1.402 KPa/pF and then to 1.736 KPa/pF, which are calculated from Table 5, Table 11 and Table 13.

#### 3.2.3. Effect of Poisson’s Ratio on Input–Output Relationships

**Figure 24:**
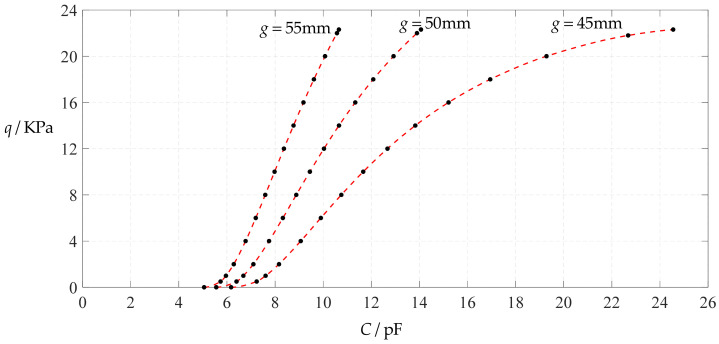
Variations of pressure *q* with capacitance *C*, when *a* = 100 mm, *h* = 1 mm, *E* = 7.84 MPa, *ν* = 0.32, *t* = 0.1 mm and *g* = 45 mm, 50 mm and 55 mm.

**Figure 25:**
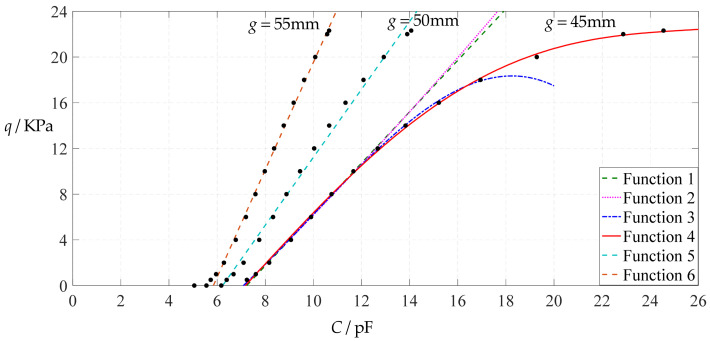
Least-squares fitting of the relationships between *q* and *C* in Figure 24.

**Figure 26:**
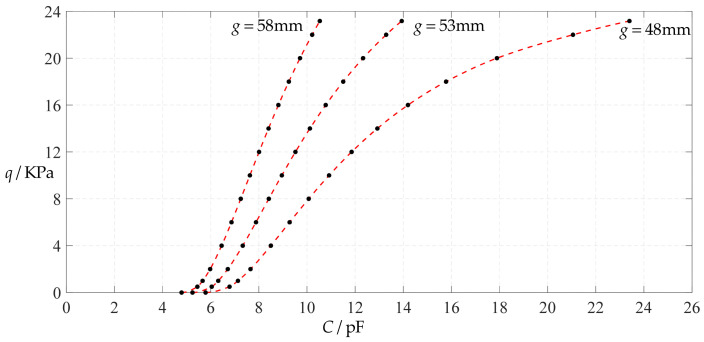
Variations of pressure *q* with capacitance *C*, when *a* = 100 mm, *h* = 1 mm, *E* = 7.84 MPa, *ν* = 0.16, *t* = 0.1 mm and *g* = 48 mm, 53 mm and 58 mm.

**Figure 27:**
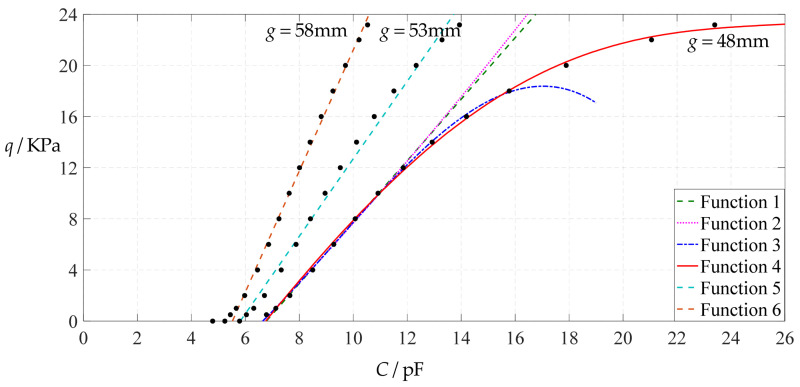
Least-squares fitting of the relationships between *q* and *C* in Figure 26.

**Figure 28:**
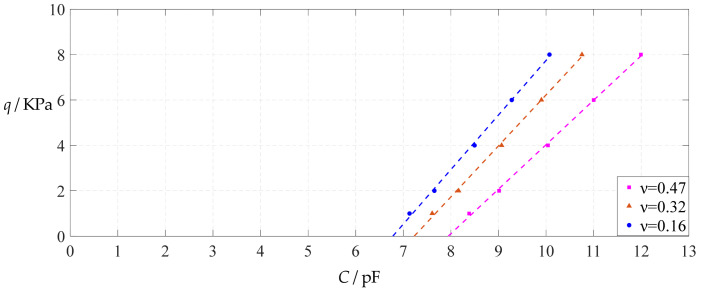
The effect of changing the Poisson’s ratio *v* on Function 1 in Table 5, Table 15 and Table 17 (fitted by a straight line).

**Figure 29:**
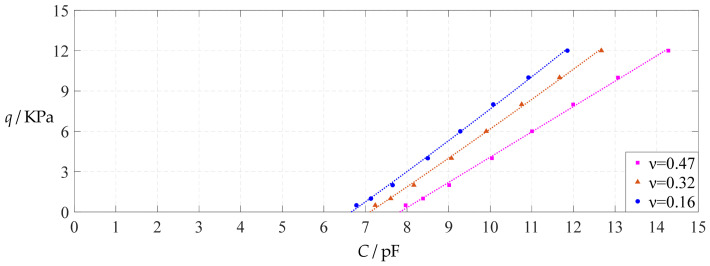
The effect of changing the Poisson’s ratio *v* on Function 2 in Table 5, Table 15 and Table 17 (fitted by a quadratic function).

**Figure 30:**
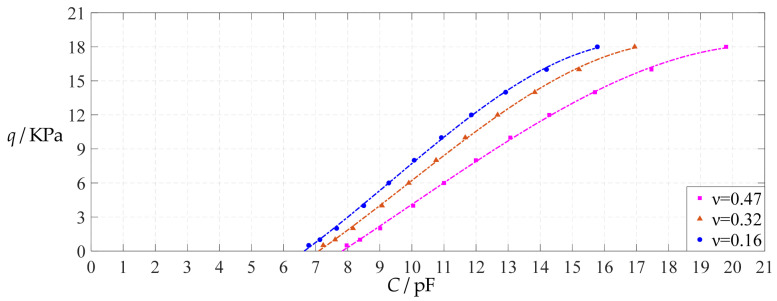
The effect of changing the Poisson’s ratio *v* on Function 3 in Table 5, Table 15 and Table 17 (fitted by a cubic function).

**Figure 31:**
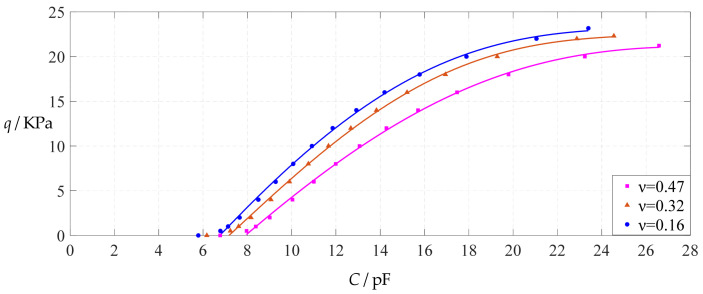
The effect of changing the Poisson’s ratio *v* on Function 4 in Table 5, Table 15 and Table 17 (fitted by a quartic function).

The design parameters used in Section 3.1 are still used as reference, that is, *v* = 0.47, *E* = 7.84 MPa, *a* = 100 mm, *h* = 1 mm, *t* = 0.1 mm, *ε*_0_ = 8.854 × 10^−12^ F/m = 8.854 × 10^−3^ pF/mm, *ε*_r1_ = 2.5, *ε*_r2_ = 1.00053, *σ*_y_ = 2.4 MPa and *σ*_m_ ≤ 0.7 *σ*_y_ ≈ 1.68 MPa. In this section, the Poisson’s ratio *v* of the conductive membrane is first decreased from the reference value of 0.47 (for such as polymer films) to 0.32 (for such as metal films) and then further decreased to 0.16 (for such as graphene films). When *v* = 0.32, the calculation results are listed in Table 14, the relationships between input capacitance *C* and output pressure *q* are shown in Figure 24, the results of least-squares fitting are shown in Figure 25, the fitting functions are listed in Table 15, and the average sum of fitting error squares of each fitting function is shown in the footer of Table 15. When *v* = 0.16, the calculation results are listed in Table 16, the input–output relationships are shown in Figure 26, the results of least-squares fitting are shown in Figure 27, the fitting functions are listed in Table 17, and the average sum of fitting error squares of each fitting function is shown in the footer of Table 17. The effects of a decrease in the Poisson’s ratio *v* from 0.47 to 0.32 and then to 0.16 on the fitting functions (Functions 1–4) are summarized in Figure 28, Figure 29, Figure 30 and Figure 31. 

As can be seen from Figure 28, Figure 29, Figure 30 and Figure 31, especially from Figure 31, the change of the Poisson’s ratio *v* from 0.47 to 0.32 and then to 0.16 results in only a small nearly parallel shift of the *q*(*C*) curves along the horizontal coordinate axis; that is, such a large change in the Poisson’s ratio *v* from 0.47 to 0.32 and then to 0.16 does not have much effect on both the range of output pressure *q* and the range of input capacitance *C*. This means that when choosing a polymer conductive membrane as the movable electrode plate of a capacitor in a non-touch mode circular capacitive pressure sensor, it is sufficient to know the approximate range of Poisson’s ratio rather than its exact value. 

#### 3.2.4. Effect of Insulator Layer Thickness on Input–Output Relationships

**Figure 32:**
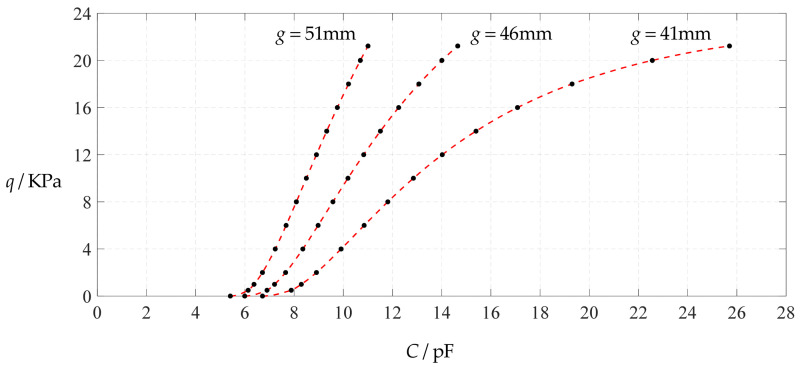
Variations of pressure *q* with capacitance *C*, when *a* = 100 mm, *h* = 1 mm, *E* = 7.84 MPa, *ν* = 0.47, *t* = 1 mm and *g* = 41 mm, 46 mm and 51 mm.

**Figure 33:**
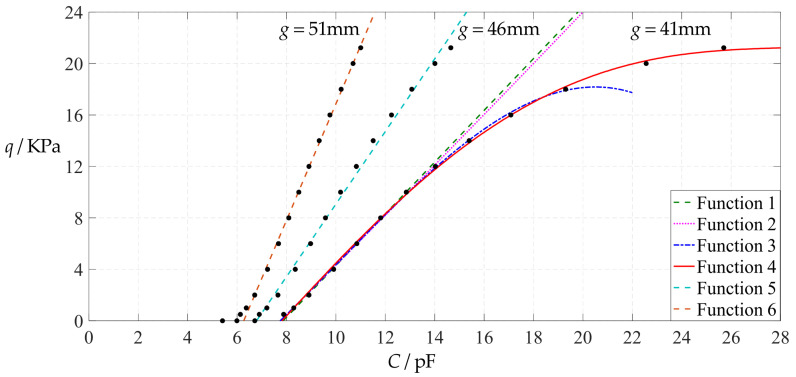
Least-squares fitting of the relationships between *q* and *C* in Figure 32.

**Figure 34:**
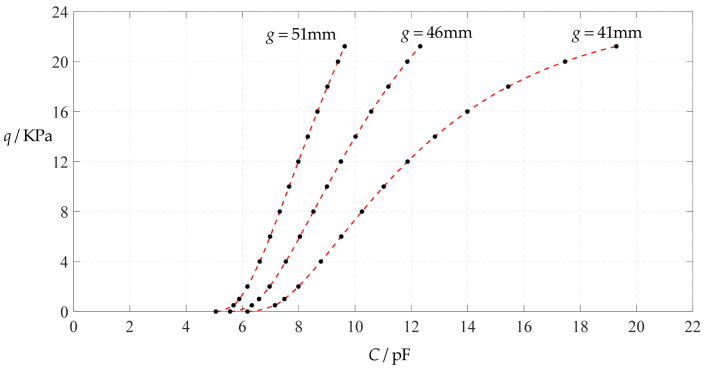
Variations of pressure *q* with capacitance *C*, when *a* = 100 mm, *h* = 1 mm, *E* = 7.84 MPa, *ν* = 0.47, *t* = 10 mm and *g* = 41 mm, 46 mm and 51 mm.

**Figure 35:**
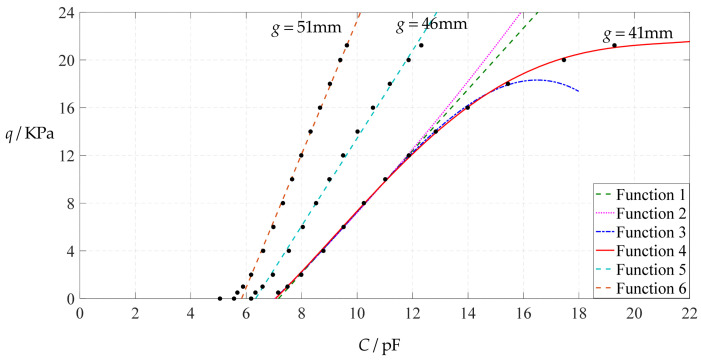
Least-squares fitting of the relationships between *q* and *C* in Figure 34.

**Figure 36:**
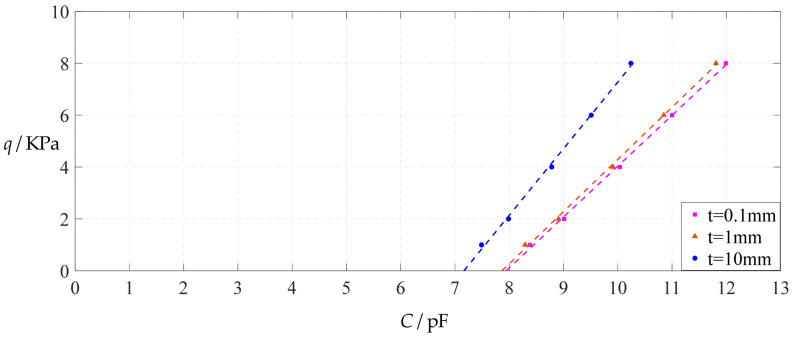
The effect of changing the insulator layer thickness *t* on Function 1 in Table 5, Table 19 and Table 21 (fitted by a straight line).

**Figure 37:**
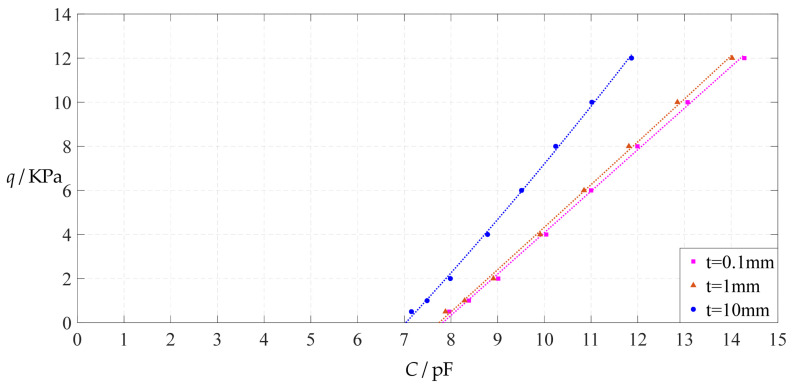
The effect of changing the insulator layer thickness *t* on Function 2 in Table 5, Table 19 and Table 21 (fitted by a quadratic function).

**Figure 38:**
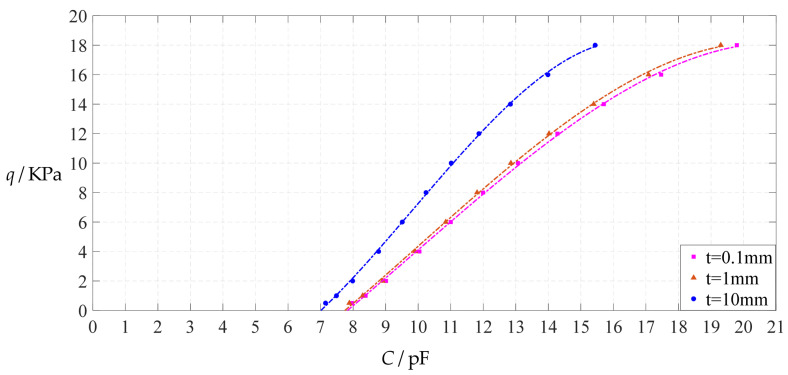
The effect of changing the insulator layer thickness *t* on Function 3 in Table 5, Table 19 and Table 21 (fitted by a cubic function).

**Figure 39:**
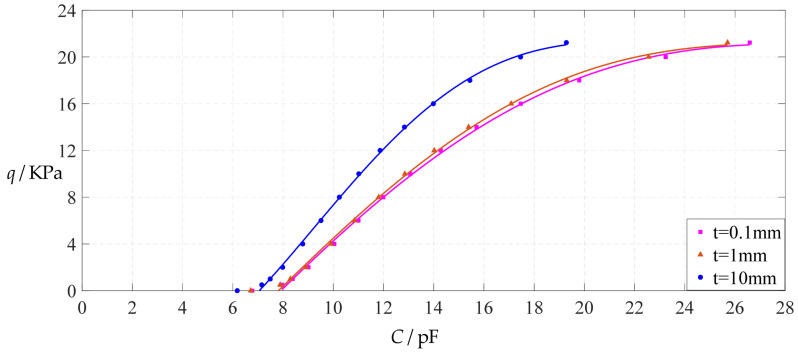
The effect of changing the insulator layer thickness *t* on Function 4 in Table 5, Table 19 and Table 21 (fitted by a quartic function).

The design parameters used in Section 3.1 are still used as reference, that is, *v* = 0.47, *E* = 7.84 MPa, *a* = 100 mm, *h* = 1 mm, *t* = 0.1 mm, *ε*_0_ = 8.854 × 10^−12^ F/m = 8.854 × 10^−3^ pF/mm, *ε*_r1_ = 2.5, *ε*_r2_ = 1.00053, *σ*_y_ = 2.4 MPa and *σ*_m_ ≤ 0.7 *σ*_y_ ≈ 1.68 MPa. In this section, the thickness *t* of the insulator layer is first increased from the reference value of 0.1 mm to 1 mm and then to 10 mm. When *t* = 1 mm, the calculation results are listed in Table 18, the relationships between input capacitance *C* and output pressure *q* are shown in Figure 32, the results of least-squares fitting are shown in Figure 33, the fitting functions are listed in Table 19, and the average sum of fitting error squares of each fitting function are shown in the footer of Table 19. When *t* = 10 mm, the calculation results are listed in Table 20, the input–output relationships are shown in Figure 34, the results of least-squares fitting are shown in Figure 35, the fitting functions are listed in Table 21, and the average sum of fitting error squares of each fitting function are shown in the footer of Table 21. The effects of an increase in the thickness *t* of the insulator layer from 0.1 mm to 1 mm and then to 10 mm on the fitting functions (Functions 1–4) are summarized in Figure 36, Figure 37, Figure 38 and Figure 39. 

From Figure 36, Figure 37, Figure 38 and Figure 39, it can be seen that increasing the thickness *t* of the insulator layer has no effect on the range of output pressure *q*, and it only reduces the range of input capacitance *C*, resulting in an increase in the output pressure per unit capacitance. Taking Function 4 as an example, when the thickness *t* of the insulator layer increases from 0.1 mm to 10 mm, the output pressure per unit capacitance increases from 1.071 KPa/pF (calculated from Table 5) to 1.620 KPa/pF (calculated from Table 21). As a result, it is generally welcome for the thickness *t* of the insulator layer to be as thin as possible.

## 4. Concluding Remarks

In this paper, an analytical solution-based method for the design and numerical calibration of polymer conductive membrane-based non-touch mode circular capacitive pressure sensors is presented. This novel method can provide effective theoretical support for the design and fabrication of such sensors. From this study, the following conclusions can be drawn.

The so-called nearly linear input–output relationships of non-touch mode capacitive pressure sensors can be easily realized by using the presented analytical solution-based method. It can be seen from Section 3 that the desired nearly linear input–output relationships can be easily achieved by changing design parameters, such as membrane thickness, Young’s modulus of elasticity and the initial gap between the initially flat undeflected conductive membrane and the insulator layer coating on the substrate electrode plate. Therefore, the view in the literature is open to debate that non-touch mode capacitive pressure sensors are far inferior to touch mode capacitive pressure sensors in the easy realization of nearly linear input–output relationships.

The change in membrane thickness has no effect on the range of input capacitance and only affects the range of output pressure, which increases with the increase in membrane thickness.

The change in Young’s modulus of elasticity affects both the range of output pressure and the range of input capacitance, where the range of output pressure increases with the decrease in Young’s modulus of elasticity, and the range of input capacitance decreases with the decrease in Young’s modulus of elasticity. 

The change in Poisson’s ratio has a very limited effect on input–output relationships. Therefore, it is sufficient to know the approximate range of Poisson’s ratio rather than its exact value when choosing a polymer conductive membrane as the movable electrode plate of a capacitor of a non-touch mode circular capacitive pressure sensor.

The change in insulator layer thickness has no effect on the range of output pressure and only affects the range of input capacitance, which decreases with the increase in insulator layer thickness.

## Figures and Tables

**Table 1:** The calculation results of *b*_0_ and *c*_0_, *w*_m_ and *σ*_m_ for *a* = 100 mm, *h* = 1 mm, *E* = 7.84 MPa and *ν* = 0.47.

*q*/KPa	*b*_0_	*c*_0_	*w*_m_/mm	*σ*_m_/MPa
0	0.000000	0.000000	0.000	0.000
0.5	0.015819	0.112374	11.237	0.124
1	0.025251	0.141729	14.173	0.198
2	0.040443	0.178839	17.884	0.317
4	0.065119	0.225793	22.579	0.511
6	0.086362	0.258841	25.884	0.677
8	0.105751	0.285194	28.519	0.829
10	0.123933	0.307465	30.747	0.972
12	0.141247	0.326937	32.694	1.107
14	0.157901	0.344351	34.435	1.238
16	0.174030	0.360175	36.018	1.364
18	0.189729	0.374732	37.473	1.487
20	0.205068	0.388252	38.825	1.608
21.225	0.214308	0.396696	39.670	1.680
22	0.220099	0.400906	40.091	1.726
24	0.234862	0.412826	41.283	1.841
26	0.249389	0.424116	42.412	1.955
28	0.263707	0.434859	43.486	2.067
30	0.277838	0.445123	44.512	2.178
32	0.291798	0.454965	45.496	2.288
34	0.305603	0.464432	46.443	2.396

**Table 2:** The calculation results of the coefficients *c*_2*i*_ (*i* = 0, 1, 2, 3) for *a* = 100 mm, *h* = 1 mm, *E* = 7.84 MPa and *ν* = 0.47.

*q*/KPa	*c*_0_	*c*_2_	*c*_4_	*c*_6_
0	0.000000	0.000000	0.000000	0.000000
0.5	0.112374	−0.100790	−0.009047	−1.854564 × 10^−3^
1	0.141729	−0.126281	−0.011851	−2.564440 × 10^−3^
2	0.178839	−0.157694	−0.015787	−3.678232 × 10^−3^
4	0.225793	−0.195873	−0.021459	−5.497780 × 10^−3^
6	0.258841	−0.221541	−0.025922	−7.084793 × 10^−3^
8	0.285194	−0.241228	−0.029749	−8.541169 × 10^−3^
10	0.307465	−0.257299	−0.033155	−9.905430 × 10^−3^
12	0.326937	−0.270910	−0.036253	−1.119777 × 10^−2^
14	0.344351	−0.282727	−0.039109	−1.243081 × 10^−2^
16	0.360175	−0.293170	−0.041768	−1.361335 × 10^−2^
18	0.374732	−0.302525	−0.044262	−1.475200 × 10^−2^
20	0.388252	−0.310997	−0.046617	−1.585194 × 10^−2^
21.225	0.396696	−0.315815	−0.047998	−1.650838 × 10^−2^

**Table 3:** The calculation results of the coefficients *c*_2*i*_ (*i* = 4, 5, 6, 7) for *a* = 100 mm, *h* = 1 mm, *E* = 7.84 MPa and *ν* = 0.47.

*q*/KPa	*c*_8_	*c*_10_	*c*_12_	*c*_14_
0	0.000000	0.000000	0.000000	0.000000
0.5	−4.789369 × 10^−4^	−1.389200 × 10^−4^	−4.326591 × 10^−5^	−1.414301 × 10^−5^
1	−7.036597 × 10^−4^	−2.176087 × 10^−4^	−7.241011 × 10^−5^	−2.532468 × 10^−5^
2	−1.094379 × 10^−3^	−3.682477 × 10^−4^	−1.335929 × 10^−4^	−5.100339 × 10^−5^
4	−1.810971 × 10^−3^	−6.767557 × 10^−4^	−2.731357 × 10^−4^	−1.161416 × 10^−4^
6	−2.497975 × 10^−3^	−1.000748 × 10^−3^	−4.333885 × 10^−4^	−1.978624 × 10^−4^
8	−3.169617 × 10^−3^	−1.337792 × 10^−3^	−6.107136 × 10^−4^	−2.940391 × 10^−4^
10	−3.829440 × 10^−3^	−1.684867 × 10^−3^	−8.021195 × 10^−4^	−4.028732 × 10^−4^
12	−4.478700 × 10^−3^	−2.039522 × 10^−3^	−1.005254 × 10^−3^	−5.228583 × 10^−4^
14	−5.118047 × 10^−3^	−2.399892 × 10^−3^	−1.218271 × 10^−3^	−6.527353 × 10^−4^
16	−5.747980 × 10^−3^	−2.764584 × 10^−3^	−1.439715 × 10^−3^	−7.914557 × 10^−4^
18	−6.368975 × 10^−3^	−3.132569 × 10^−3^	−1.668442 × 10^−3^	−9.381483 × 10^−4^
20	−6.981522 × 10^−3^	−3.503093 × 10^−3^	−1.903546 × 10^−3^	−1.092091 × 10^−3^
21.225	−7.352749 × 10^−3^	−3.731051 × 10^−3^	−2.050382 × 10^−3^	−1.189700 × 10^−3^

**Table 4:** The calculation results for *a* = 100 mm, *h* = 1 mm, *E* = 7.84 MPa, *ν* = 0.47, *t* = 0.1 mm, and *g* = 41 mm, 46 mm and 51 mm.

*q*/KPa	*w_m_*/mm	*σ_m_*/MPa	*C*/pF		
			*g* = 41 mm	*g* = 46 mm	*g* = 51 mm
0	0	0	6.775	6.039	5.447
0.5	11.237	0.124	7.965	6.961	6.182
1	14.173	0.198	8.384	7.273	6.424
2	17.884	0.317	9.013	7.730	6.772
4	22.579	0.511	10.040	8.446	7.301
6	25.884	0.677	11.002	9.081	7.753
8	28.519	0.829	11.993	9.698	8.178
10	30.747	0.972	13.068	10.326	8.594
12	32.694	1.107	14.281	10.982	9.012
14	34.435	1.238	15.707	11.683	9.439
16	36.018	1.364	17.468	12.448	9.883
18	37.473	1.487	19.794	13.298	10.349
20	38.825	1.608	23.239	14.266	10.843
21.225	39.670	1.680	26.585	14.935	11.164

**Table 5:** The range of pressure *q* and capacitance *C*, and the analytical expressions of Functions 1–6 in Figure 7.

Functions	*q*/KPa	*C*/pF	Functional Expressions
Function 1	1~8	8.384~11.993	*q* = −15.57 + 1.960*C*
Function 2	0.5~12	7.965~14.281	*q* = −14.59 + 1.856*C* − 0.001137*C*^2^
Function 3	0.5~18	7.965~19.794	*q* = −9.867 + 0.3584*C* + 0.1562*C*^2^ − 0.005222*C*^3^
Function 4	0~21.225	6.775~26.585	*q* = −16.64 + 1.865*C* + 0.06435*C*^2^ − 0.004878*C*^3^ + 0.00006859*C*^4^
Function 5	1~21.225	7.273~14.935	*q* = −18.73 + 2.743*C*
Function 6	1~21.225	6.424~11.164	*q* = −27.93 + 4.421*C*

Note: The average sum of fitting error squares of Functions 1–6 is 0.0088, 0.0259, 0.0233, 0.0481, 0.2590 and 0.0626, respectively.

**Table 6:** The calculation results for *a* = 100 mm, *h* = 1.5 mm, *E* = 7.84 MPa, *ν* = 0.47, *t* = 0.1 mm, and *g* = 41 mm, 46 mm and 51 mm.

*q*/KPa	*w_m_*/mm	*σ_m_*/MPa	*C*/pF		
			*g* = 41 mm	*g* = 46 mm	*g* = 51 mm
0	0.000	0.000	6.775	6.039	5.447
0.5	9.812	0.094	7.782	6.822	6.074
1	12.373	0.151	8.120	7.077	6.273
2	15.608	0.241	8.612	7.440	6.553
4	19.699	0.386	9.373	7.986	6.963
6	22.579	0.511	10.040	8.446	7.301
8	24.877	0.624	10.682	8.874	7.607
10	26.820	0.729	11.327	9.287	7.897
12	28.519	0.829	11.993	9.698	8.178
14	30.040	0.925	12.697	10.114	8.455
16	31.422	1.018	13.453	10.540	8.732
18	32.694	1.107	14.281	10.982	9.012
20	33.874	1.195	15.202	11.443	9.295
22	34.978	1.281	16.249	11.930	9.585
24	36.018	1.364	17.468	12.448	9.883
25	36.516	1.406	18.164	12.720	10.035
26	37.001	1.447	18.933	13.004	10.191
27	37.373	1.487	19.794	13.298	10.349
28	37.934	1.528	20.772	13.606	10.510
29	38.185	1.568	21.902	13.928	10.675
30	38.825	1.608	23.239	14.266	10.843
31.84	39.611	1.680	26.591	14.936	11.164

**Table 7:** The range of pressure *q* and capacitance *C*, and the analytical expressions of the Functions 1–6 in Figure 9.

Functions	*q*/KPa	*C*/pF	Functional Expressions
Function 1	1~12	8.120~11.993	*q* = −22.81 + 2.889*C*
Function 2	0.5~18	7.782~14.281	*q* = −19.88 + 2.425*C* + 0.01752*C*^2^
Function 3	0.5~27	7.782~19.794	*q* = −12.73 + 0.08131*C* + 0.2674*C*^2^ − 0.008633*C*^3^
Function 4	0~31.84	6.775~26.591	*q* = −22.87 + 2.312*C* + 0.1379*C*^2^ − 0.008860*C*^3^ + 0.0001237*C*^4^
Function 5	1~31.84	7.077~14.936	*q* = −28.34 + 4.146*C*
Function 6	1~31.84	6.273~11.164	*q* = −41.83 + 6.632*C*

Note: The average sum of fitting error squares of Functions 1–6 is 0.0393, 0.0715, 0.0614, 0.0958, 0.4674 and 0.1774, respectively.

**Table 8:** The calculation results for *a* = 100 mm, *h* = 2 mm, *E* = 7.84 MPa, *ν* = 0.47, *t* = 0.1 mm, and *g* = 41 mm, 46 mm and 51 mm.

*q*/KPa	*w_m_*/mm	*σ_m_*/MPa	*C*/pF		
			*g* = 41 mm	*g* = 46 mm	*g* = 51 mm
0	0.000	0.000	6.775	6.039	5.447
0.5	8.913	0.078	7.673	6.739	6.008
1	11.237	0.124	7.965	6.961	6.182
2	14.173	0.198	8.384	7.273	6.424
4	17.884	0.317	9.013	7.730	6.772
6	20.496	0.419	9.545	8.106	7.052
8	22.579	0.511	10.040	8.446	7.301
10	24.342	0.596	10.522	8.769	7.533
12	25.884	0.677	11.002	9.081	7.753
14	27.264	0.755	11.491	9.390	7.967
16	28.519	0.829	11.993	9.698	8.178
18	29.674	0.901	12.517	10.010	8.386
20	30.747	0.972	13.068	10.326	8.594
22	31.750	1.040	13.653	10.649	8.802
24	32.694	1.107	14.281	10.982	9.012
26	33.587	1.173	14.961	11.325	9.224
28	34.435	1.238	15.707	11.683	9.439
30	35.594	1.302	16.535	12.056	9.659
32	36.018	1.364	17.468	12.448	9.883
34	36.760	1.426	18.538	12.860	10.113
36	37.473	1.487	19.794	13.298	10.349
38	38.161	1.548	21.315	13.765	10.592
40	38.825	1.608	23.239	14.266	10.843
42	39.468	1.667	25.847	14.807	11.104
42.45	39.610	1.680	26.585	14.935	11.164

**Table 9:** The range of pressure *q* and capacitance *C*, and the analytical expressions of Functions 1–6 in Figure 11.

Functions	*q*/KPa	*C*/pF	Functional Expressions
Function 1	1~16	7.965~11.993	*q* = −30.00 + 3.813*C*
Function 2	1~24	7.965~14.281	*q* = −29.645 + 3.7645*C* + 0.001365*C*^2^
Function 3	1~36	7.965~19.794	*q* = −21.30 + 1.011*C* + 0.2956*C*^2^ − 0.01017*C*^3^
Function 4	0~42.45	6.775~26.585	*q* = −30.96 + 2.917*C* + 0.2205*C*^2^ − 0.01388*C*^3^ + 0.0002010*C*^4^
Function 5	1~42.45	6.961~14.935	*q* = −37.33 + 5.481*C*
Function 6	1~42.45	6.182~11.164	*q* = −55.36 + 8.791*C*

Note: The average sum of fitting error squares of Functions 1–6 is 0.0991, 0.0915, 0.0854, 0.0987, 0.9849 and 0.4131, respectively.

**Table 10:** The calculation results for *a* = 100 mm, *h* = 1 mm, *E* = 5 MPa, *ν* = 0.47, *t* = 0.1 mm and *g* = 50 mm, 55 mm and 60 mm.

*q*/KPa	*w_m_*/mm	*σ_m_*/MPa	*C*/pF		
			*g* = 50 mm	*g* = 55 mm	*g* = 60 mm
0	0.000	0.000	5.556	5.051	4.631
0.5	13.063	0.107	6.478	5.798	5.248
1	16.481	0.171	6.799	6.050	5.452
2	20.804	0.275	7.277	6.419	5.745
4	26.274	0.445	8.048	6.995	6.192
6	30.121	0.593	8.756	7.503	6.575
8	33.185	0.729	9.467	7.992	6.934
10	35.774	0.857	10.217	8.485	7.286
12	38.036	0.980	11.035	8.996	7.639
14	40.061	1.099	11.954	9.537	8.000
16	41.904	1.214	13.020	10.119	8.375
18	43.603	1.326	14.305	10.757	8.769
20	45.186	1.437	15.932	11.471	9.187
22	46.673	1.545	18.158	12.284	9.637
24	48.079	1.652	21.659	13.235	10.127
24.54	48.447	1.680	23.062	13.523	10.267

**Table 11:** The range of pressure *q* and capacitance *C*, and the analytical expressions of the fitting functions in Figure 17.

Functions	*q*/KPa	*C*/pF	Functional Expressions
Function 1	1~10	6.799~10.217	*q* = −17.349 + 2.672*C*
Function 2	0.5~14	6.478~11.954	*q* = −16.73 + 2.644*C* − 0.004760*C*^2^
Function 3	0.5~20	6.478~15.932	*q* = −9.282 − 0.03216*C* + 0.3101*C*^2^ − 0.01213*C*^3^
Function 4	0~24.54	5.556~23.062	*q* = −18.81 + 2.632*C* + 0.09652*C*^2^ − 0.009415*C*^3^ − 0.0001647*C*^4^
Function 5	1~24.54	6.050~13.523	*q* = −18.10 + 3.262*C*
Function 6	1~24.54	5.452~10.267	*q* = −26.97 + 5.075*C*

Note: The average sum of fitting error squares of Functions 1–6 is 0.0123, 0.0375, 0.0273, 0.0634, 0.678 and 0.129, respectively.

**Table 12:** The calculation results for *a* = 100 mm, *h* = 1 mm, *E* = 2.5 MPa, *ν* = 0.47, *t* = 0.1 mm and *g* = 68 mm, 73 mm and 78 mm.

*q*/KPa	*w_m_*/mm	*σ_m_*/MPa	*C*/pF		
			*g* = 68 mm	*g* = 73 mm	*g* = 78 mm
0	0.000	0.000	4.086	3.807	3.563
0.5	16.481	0.086	4.708	4.338	4.023
1	20.801	0.138	4.921	4.517	4.175
2	26.274	0.223	5.237	4.779	4.396
4	33.185	0.365	5.738	5.185	4.732
6	38.036	0.490	6.187	5.540	5.020
8	41.904	0.607	6.627	5.879	5.290
10	45.186	0.718	7.079	6.219	5.555
12	48.079	0.826	7.560	6.570	5.824
14	50.698	0.930	8.084	6.941	6.102
16	53.114	1.032	8.671	7.340	6.394
18	55.376	1.132	9.345	7.779	6.706
20	57.518	1.230	10.142	8.269	7.044
22	59.566	1.327	11.119	8.828	7.415
24	61.539	1.422	12.378	9.480	7.828
26	63.450	1.516	14.127	10.261	8.296
28	65.313	1.609	16.912	11.232	8.834
29.55	66.728	1.680	21.112	12.180	9.314

**Table 13:** The range of pressure *q* and capacitance *C*, and the analytical expressions of the fitting functions in Figure 19.

Functions	*q*/KPa	*C*/pF	Functional Expressions
Function 1	1~12	4.921~7.560	*q* = −20.16 + 4.248*C*
Function 2	0.5~16	4.708~8.671	*q* = −19.13 + 4.098*C* − 0.001996*C*^2^
Function 3	0.5~22	4.708~11.119	*q* = −6.704 − 1.874*C* + 0.9372*C*^2^ − 0.04836*C*^3^
Function 4	0~29.55	4.086~21.112	*q* = −35.72 + 9.574*C* − 0.5129*C^2^ +* 0.01150*C*^3^ − 0.00008396*C*^4^
Function 5	1~29.55	4.517~12.180	*q* = −14.94 + 3.964*C*
Function 6	1~29.55	4.175~9.314	*q* = −22.78 + 5.878*C*

Note: The average sum of fitting error squares of Functions 1–6 is 0.0206, 0.0548, 0.0332, 0.0961, 3.1043 and 1.1813, respectively.

**Table 14:** The calculation results for *a* = 100 mm, *h* = 1 mm, *E* = 7.84 MPa, *ν* = 0.32, *t* = 0.1 mm and *g* = 45 mm, 50 mm and 55 mm.

*q*/KPa	*w_m_*/mm	*σ_m_*/MPa	*C*/pF		
			*g* = 45 mm	*g* = 50 mm	*g* = 55 mm
0	0.000	0.000	6.173	5.556	5.051
0.5	12.048	0.118	7.236	6.398	5.734
1	15.196	0.189	7.607	6.682	5.959
2	19.177	0.303	8.164	7.099	6.283
4	24.212	0.488	9.067	7.750	6.775
6	27.755	0.648	9.903	8.325	7.197
8	30.579	0.795	10.754	8.882	7.592
10	32.966	0.932	11.664	9.446	7.980
12	35.054	1.064	12.672	10.033	8.369
14	36.922	1.190	13.830	10.657	8.767
16	38.623	1.312	15.211	11.334	9.181
18	40.189	1.432	16.944	12.082	9.615
20	41.647	1.548	19.283	12.926	10.077
22	43.014	1.663	22.876	13.899	10.574
22.31	43.219	1.680	24.548	14.065	10.654

**Table 15:** The range of pressure *q* and capacitance *C*, and the analytical expressions of the fitting functions in Figure 25.

Functions	*q*/KPa	*C*/pF	Functional Expressions
Function 1	1~8	7.607~10.754	*q* = −16.243 + 2.247*C*
Function 2	0.5~12	7.236~12.672	*q* = −13.84 + 1.816*C* + 0.01848*C*^2^
Function 3	0.5~18	7.236~16.944	*q* = −5.703 − 0.8907*C* + 0.3141*C*^2^ − 0.01058*C*^3^
Function 4	0~22.31	6.173~24.548	*q* = −13.26 + 0.9157*C* + 0.2073*C*^2^ − 0.01209*C*^3^ + 0.0001844*C*^4^
Function 5	1~22.31	6.682~14.065	*q* = −18.52 + 2.974*C*
Function 6	1~22.31	5.959~10.654	*q* = −27.45 + 4.697*C*

Note: The average sum of fitting error squares of Functions 1–6 is 0.0112, 0.0245, 0.0182, 0.03928, 0.3715 and 0.0729, respectively.

**Table 16:** The calculation results for *a* = 100 mm, *h* = 1 mm, *E* = 7.84 MPa, *ν* = 0.16, *t* = 0.1 mm and *g* = 48 mm, 53 mm and 58 mm.

*q*/KPa	*w_m_*/mm	*σ_m_*/MPa	*C*/pF		
			*g* = 48 mm	*g* = 53 mm	*g* = 58 mm
0	0.000	0.000	5.787	5.242	4.790
0.5	12.756	0.114	6.783	6.041	5.446
1	16.091	0.182	7.131	6.312	5.663
2	20.307	0.292	7.653	6.709	5.976
4	25.639	0.472	8.498	7.331	6.453
6	29.390	0.627	9.279	7.881	6.863
8	32.381	0.769	10.072	8.414	7.248
10	34.910	0.903	10.918	8.955	7.627
12	37.126	1.031	11.854	9.520	8.009
14	39.113	1.154	12.924	10.122	8.402
16	40.925	1.274	14.196	10.778	8.812
18	42.598	1.390	15.780	11.506	9.246
20	44.160	1.504	17.892	12.331	9.711
22	45.630	1.616	21.054	13.290	10.215
23.173	46.455	1.680	23.397	13.937	10.532

**Table 17:** The range of pressure *q* and capacitance *C*, and the analytical expressions of the fitting functions in Figure 27.

Functions	*q*/KPa	*C*/pF	Functional Expressions
Function 1	1~8	7.131~10.072	*q* = −16.29 + 2.404*C*
Function 2	0.5~12	6.783~11.854	*q* = −13.65 + 1.889*C* − 0.02428*C*^2^
Function 3	0.5~18	6.783~15.780	*q* = −5.029 − 1.161*C* + 0.3782*C*^2^ − 0.01347*C*^3^
Function 4	0~23.173	5.787~23.397	*q* = −17.29 + 2.232*C* + 0.09929*C*^2^ − 0.008501*C*^3^ − 0.0001417*C*^4^
Function 5	1~23.173	6.312~13.937	*q* = −17.62 + 3.031*C*
Function 6	1~23.173	5.663~10.532	*q* = −26.15 + 4.735*C*

Note: The average sum of fitting error squares of Functions 1–6 is 0.0117, 0.0245, 0.0177, 0.0597, 0.5367 and 0.1019, respectively.

**Table 18:** The calculation results for *a* = 100 mm, *h* = 1 mm, *E* = 7.84 MPa, *ν* = 0.47, *t* = 1 mm and *g* = 41 mm, 46 mm and 51 mm.

*q*/KPa	*w_m_*/mm	*σ_m_*/MPa	*C*/pF		
			*g* = 41 mm	*g* = 46 mm	*g* = 51 mm
0	0.000	0.000	6.716	5.992	5.409
0.5	11.237	0.124	7.884	6.899	6.133
1	14.173	0.198	8.293	7.205	6.371
2	17.884	0.317	8.909	7.654	6.713
4	22.579	0.511	9.911	8.355	7.233
6	25.884	0.677	10.848	8.976	7.676
8	28.519	0.829	11.810	9.578	8.092
10	30.747	0.972	12.850	10.189	8.499
12	32.694	1.107	14.022	10.828	8.908
14	34.435	1.238	15.394	11.509	9.325
16	36.018	1.364	17.082	12.250	9.758
18	37.473	1.487	19.300	13.073	10.212
20	38.825	1.608	22.560	14.007	10.693
21.225	39.670	1.680	25.700	14.651	11.005

**Table 19:** The range of pressure *q* and capacitance *C*, and the analytical expressions of the fitting functions in Figure 33.

Functions	*q*/KPa	*C*/pF	Functional Expressions
Function 1	1~8	8.294~11.810	*q* = −15.82 + 2.012*C*
Function 2	0.5~12	7.884~14.022	*q* = −14.56 + 1.850*C* + 0.003927*C*^2^
Function 3	0.5~18	7.884~19.300	*q* = −9.152 + 0.1343*C* + 0.1818*C*^2^ − 0.006018*C*^3^
Function 4	0~21.225	6.716~25.700	*q* = −14.44 + 1.227*C* + 0.1336*C*^2^ − 0.007816*C*^3^ + 0.0001108*C*^4^
Function 5	1~21.225	7.205~14.651	*q* = −19.15 + 2.822*C*
Function 6	1~21.225	6.371~11.005	*q* = −28.36 + 4.522*C*

Note: The average sum of fitting error squares of Functions 1–6 is 0.0093, 0.0258, 0.0226, 0.0431, 0.2428 and 0.0641, respectively.

**Table 20:** The calculation results for *a* = 100 mm, *h* = 1 mm, *E* = 7.84 MPa, *ν* = 0.47, *t* = 10 mm and *g* = 41 mm, 46 mm and 51 mm.

*q*/KPa	*w_m_*/mm	*σ_m_*/MPa	*C*/pF		
			*g* = 41 mm	*g* = 46 mm	*g* = 51 mm
0	0.000	0.000	6.178	5.561	5.055
0.5	11.237	0.124	7.154	6.333	5.682
1	14.173	0.198	7.489	6.590	5.886
2	17.884	0.317	7.987	6.964	6.176
4	22.579	0.511	8.784	7.539	6.613
6	25.884	0.677	9.512	8.041	6.982
8	28.519	0.829	10.243	8.521	7.325
10	30.747	0.972	11.017	9.002	7.656
12	32.694	1.107	11.867	9.496	7.986
14	34.435	1.238	12.836	10.016	8.321
16	36.018	1.364	13.988	10.573	8.663
18	37.473	1.487	15.441	11.181	9.019
20	38.825	1.608	17.460	11.857	9.393
21.225	39.670	1.680	19.283	12.315	9.632

**Table 21:** The range of pressure *q* and capacitance *C*, and the analytical expressions of the fitting functions in Figure 35.

Functions	*q*/KPa	*C*/pF	Functional Expressions
Function 1	1~8	7.489~10.243	*q* = −18.39 + 2.566*C*
Function 2	0.5~12	7.154~11.867	*q* = −13.80 + 1.634*C* − 0.04658*C*^2^
Function 3	0.5~18	7.154~15.441	*q* = 0.3096 − 3.154*C* + 0.5811*C*^2^ − 0.01963*C*^3^
Function 4	0~21.225	6.178~19.283	*q* = 0.005001 − 3.930*C* + 0.8241*C*^2^ − 0.04285*C*^3^ + 0.0007059*C*^4^
Function 5	1~21.225	6.590~12.315	*q* = −23.29 + 3.673*C*
Function 6	1~21.225	5.886~9.632	*q* = −32.63 + 5.593*C*

Note: The average sum of fitting error squares of Functions 1–6 is 0.0150, 0.0251, 0.0168, 0.0281, 0.1352 and 0.0876, respectively.

**Figure A1:**
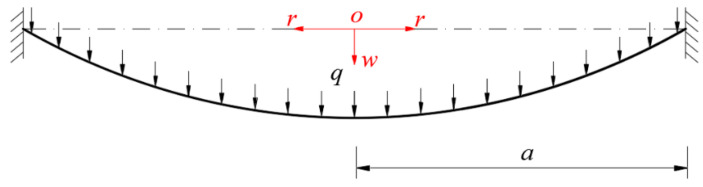
Sketch of the circular membrane under loads *q*.

**Figure A2:**
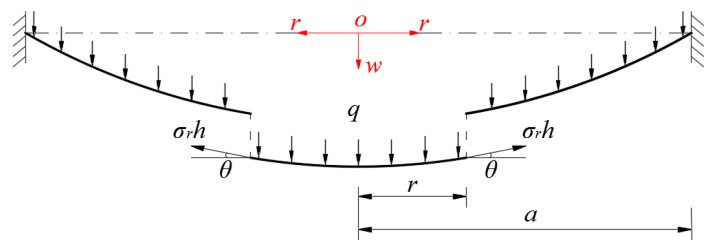
Sketch of a free body with radius 0 *≤ r ≤ a*.

## Data Availability

Not applicable.

## Appendix A

A peripherally fixed, initially flat and taut linearly elastic circular membrane with Young’s modulus of elasticity *E*, Poisson’s ratio *ν*, thickness *h*, and radius *a* is subjected to a uniformly distributed transverse loads *q*, as shown in Figure A1, where *r* is the radial coordinate, *w* is the transversal displacement, *o* is and the original point of the introduced cylindrical coordinates system (*r*, *ϕ*, *w*) (where the polar coordinate plane (*r*, *ϕ*) is located in the plane in which the geometric middle plane of the initially flat circular membrane is located). Let us take a free body with radius 0 ≤ *r* ≤ *a* from the deflected circular membrane under uniformly distributed transverse loads *q*, as shown in Figure A2, to study its static problem of equilibrium.

In the vertical direction perpendicular to the initially flat circular membrane, there are two vertical forces acting the free body, that is, the *πr^2^q* produced by the loads *q* within *r*, and the *2πrσ_r_h*sin*θ* produced by the membrane force *σ_r_h*, where *σ_r_* is radial stress. So, the out-of-plane equilibrium condition is

(A1) $$2\pi r\sigma_{r}h\sin\theta =\pi r^{2}q,$$

where

(A2) $$\sin\theta =1/\sqrt{1+1/\tan^{2}\theta }=1/\sqrt{1+1/{\left(-dw/dr\right)}^{2}}.$$

Substituting Equation (A2) into Equation (A1) yields

(A3) $$\frac{1}{2}rq\sqrt{1+1/{\left(dw/dr\right)}^{2}}=\sigma_{r}h.$$

While in the direction parallel to the initially flat circular membrane, the equilibrium condition may be written as [36]

(A4) $$\frac{d(r\sigma_{r})}{dr}-\sigma_{t}[1+{\left(-\frac{dw}{dr}\right)}^{2}]=0,$$

where *σ**_t_* denotes circumferential stress. The derivation of Equation (A4) is detailed in [36]. If the radial and circumferential strain and the radial displacement are denoted by *e_r_*, *e_t_* and *u*, respectively, then the relationships between strain and displacement for large deflection problems may be written as [37]

(A5) $$e_{r}={\left[{\left(1+\frac{du}{dr}\right)}^{2}+{\left(\frac{dw}{dr}\right)}^{2}\right]}^{1/2}-1$$

and

(A6) $$e_{t}=\frac{u}{r}.$$

Moreover, the relationships between stress and strain are still assumed to satisfy linear elasticity and expressed in terms of generalized Hooke’s law [38]

(A7) $$\sigma_{r}=\frac{E}{1-\nu^{2}}(e_{r}+\nu e_{t})$$

and

(A8) $$\sigma_{t}=\frac{E}{1-\nu^{2}}(e_{t}+\nu e_{r}).$$

Substituting Equations (A5) and (A6) into Equations (A7) and (A8) yields

(A9) $$\sigma_{r}=\frac{E}{1-\nu^{2}}\{{\left[{\left(1+\frac{du}{dr}\right)}^{2}+{\left(\frac{dw}{dr}\right)}^{2}\right]}^{1/2}-1+\nu \frac{u}{r}\}$$

and

(A10) $$\sigma_{t}=\frac{E}{1-\nu^{2}}\{\frac{u}{r}+\nu {\left[{\left(1+\frac{du}{dr}\right)}^{2}+{\left(\frac{dw}{dr}\right)}^{2}\right]}^{1/2}-\nu \}.$$

By means of Equations (A4), (A9) and (A10), one has

(A11) $$\frac{u}{r}=\frac{1}{E}(\sigma_{t}-\nu \sigma_{r})=\frac{1}{E}[\frac{\frac{d(r\sigma_{r})}{dr}}{1+{\left(-\frac{dw}{dr}\right)}^{2}}-\nu \sigma_{r}].$$

After substituting the *u* in Equation (A11) into Equation (A9), we obtain an equation containing only the radial stress *σ_r_* and deflection *w*(*r*)

(A12) $$\begin{array}{l} {\left\{1+\frac{1}{E}\frac{\frac{d(r\sigma_{r})}{dr}}{1+{\left(-\frac{dw}{dr}\right)}^{2}}-\frac{\nu \sigma_{r}}{E}+\frac{r}{E}\frac{d}{dr}[\frac{\frac{d(r\sigma_{r})}{dr}}{1+{\left(-\frac{dw}{dr}\right)}^{2}}]-\frac{r\nu }{E}\frac{d\sigma_{r}}{dr}\right\}}^{2}+{\left(\frac{dw}{dr}\right)}^{2}\\ -{\left[\frac{\sigma_{r}}{E}-\frac{\nu }{E}\frac{\frac{d(r\sigma_{r})}{dr}}{1+{\left(-\frac{dw}{dr}\right)}^{2}}+1\right]}^{2}=0 \end{array}.$$

Equations (A3) and (A12) are two equations for solving the radial stress *σ_r_* and deflection *w*(*r*). The boundary conditions, under which the particular solutions of the radial stress *σ_r_* and deflection *w*(*r*) can be determined, are

(A13) w=0 at r=a,

(A14) u=0 at r=a

and

(A15) $$\frac{dw}{dr}=0\;\mathrm{at}\;r=0.$$

Let us introduce the following dimensionless variables

(A16) $$Q=\frac{qa}{Eh},W=\frac{w}{a},S_{r}=\frac{\sigma_{r}}{E},S_{t}=\frac{\sigma_{t}}{E},x=\frac{r}{a},\alpha =\frac{b}{a},$$

and transform Equations (A3), (A12), (A13)–(A15) into

(A17) $$(4S_{r}^{2}-x^{2}Q^{2}){\left(-\frac{dW}{dx}\right)}^{2}-x^{2}Q^{2}=0,$$

(A18) $$\begin{array}{l} {\left\{1+\frac{\frac{d(xS_{r})}{dx}}{1+{\left(-\frac{dW}{dx}\right)}^{2}}-\nu S_{r}+x\frac{d}{dx}[\frac{\frac{d(xS_{r})}{dx}}{1+{\left(-\frac{dW}{dx}\right)}^{2}}]-x\nu \frac{dS_{r}}{dx}\right\}}^{2}+{\left(\frac{dW}{dx}\right)}^{2}\\ -{\left[S_{r}-\nu \frac{\frac{d(xS_{r})}{dx}}{1+{\left(-\frac{dW}{dx}\right)}^{2}}+1\right]}^{2}=0 \end{array},$$

(A19) W=0 at x=1,

(A20) $$S_{t}-\nu S_{r}=\frac{\frac{d(xS_{r})}{dx}}{1+{\left(-\frac{dW}{dx}\right)}^{2}}-\nu S_{r}=0\;\mathrm{at}\;x=1$$

and

(A21) $$\frac{dW}{dx}=0\;\mathrm{at}\;x=0.$$

Since the values of stress and deflection are both finite at *x* = 0, *S_r_* and *W* can be expanded into the power series of the *x*, i.e., letting

(A22) $$S_{r}=\sum_{i=0}^{\infty }b_{i}x^{i},$$

and

(A23) $$W=\sum_{i=0}^{\infty }c_{i}x^{i}.$$

After substituting Equations (A22) and (A23) into Equations (A17) and (A18), it is found that $b_{i}\equiv 0$ and $c_{i}\equiv 0$ when *i* is odd, and when *i* is even, $b_{i}$ and $c_{i}$ can be expressed into the polynomial of the first coefficient $b_{0}$, which are listed in Appendix B. The remaining two coefficients, $b_{0}$ and $c_{0}$, are often called undetermined coefficients, which can be determined by using the boundary conditions Equations (A19) and (A20). From Equations (A22) and (A23), Equation (A20) gives

(A24) $$(1-\nu )\sum_{i=0}^{\infty }b_{i}+\sum_{i=1}^{\infty }ib_{i}-\nu \sum_{i=0}^{\infty }b_{i}{\left(-\sum_{i=1}^{\infty }ic_{i}\right)}^{2}=0,$$

and from Equation (A23), Equation (A19) gives

(A25) $$c_{0}=-\sum_{i=1}^{\infty }c_{i}.$$

After substituting all expressions of $b_{i}$ and $c_{i}$ (*i* = 2, 4, 6, …) in Appendix B into Equation (A24), an equation which contains only the undetermined constant $b_{0}$ can be obtained. Therefore, the undetermined constant $b_{0}$ can be determined by solving this univariate variable equation. So, with the known $b_{0}$, all the coefficients $c_{i}$ (*i* = 2, 4, 6, …) can be determined, and the undetermined constant $c_{0}$ can thus be determined by Equation (A25). The problem under consideration is thus solved.

## Appendix B

$$b_{2}=\frac{1}{64}\frac{Q^{2}[(2v^{2}+4v-6)b_{0}^{2}+(-2v-6)b_{0}+1]}{(vb_{0}-b_{0}-1)b_{0}^{2}},$$

$$\begin{array}{l} b_{4}=\frac{Q^{4}}{12288{\left(vb_{0}-b_{0}-1\right)}^{3}b_{0}^{5}}[(4v^{5}+20v^{4}-24v^{3}-88v^{2}+148v-60)b_{0}^{5}+(-12v^{4}\\ \;-72v^{3}+264v-180)b_{0}^{4}+(4v^{3}+108v^{2}+60v-172)b_{0}^{3}+(6v^{2}-64v-38)b_{0}^{2}\\ \;+(-7v+21)b_{0}+2] \end{array},$$

$$\begin{array}{l} b_{6}=-\frac{Q^{6}}{4718592b_{0}^{8}{\left(\nu b_{0}-b_{0}-1\right)}^{5}}[(48\nu^{8}+336\nu^{7}-432\nu^{6}-2544\nu^{5}+4080v^{4}\\ \;+3312v^{3}-10896v^{2}+8812v-2016)b_{0}^{8}+(-240\nu^{7}-1920\nu^{6}+240\nu^{5}\\ \;+12960v^{4}-7440v^{3}-24000v^{2}+30480v-10080)b_{0}^{7}+(412\nu^{6}+5696\nu^{5}\\ \;+396v^{4}-20704v^{3}-3404v^{2}+36384v-18780)b_{0}^{6}+(-440v^{5}-9400v^{4}\\ \;-432v^{3}+16016v^{2}+9064v-14808)b_{0}^{5}+(196v^{4}+10044v^{3}-396v^{2}\\ \;-7084v-2760)b_{0}^{4}+(64v^{3}-6508v^{2}+328v+1508)b_{0}^{3}+(-139v^{2}\\ \;+2492v-365)b_{0}^{2}+(70v-414)b_{0}-13] \end{array},$$

$$\begin{array}{l} b_{8}=-\frac{Q^{8}}{3019898880b_{0}^{11}{\left(\nu b_{0}-b_{0}-1\right)}^{7}}[(3360\nu^{10}+24960\nu^{9}-80160\nu^{8}-199680\nu^{7}\\ \;+840000\nu^{6}-349440\nu^{5}-2103360\nu^{4}+4085760\nu^{3}-3354720\nu^{2}+1353600v\\ \;-220320)b_{0}^{11}+(-23520\nu^{9}-198240\nu^{8}+362880\nu^{7}+1760640\nu^{6}-4119360\nu^{5}\\ \;-1673280\nu^{4}+13050240v^{3}-15550080v^{2}+7932960v-1542240)b_{0}^{10}\\ \;+(1144\nu^{9}+10392\nu^{8}+972096\nu^{7}-998912\nu^{6}-5469840\nu^{5}+7437936v^{4}\\ \;+10223488v^{3}-26362176v^{2}+18746712v-4560840)b_{0}^{9}+(3536\nu^{8}\\ \;+159280\nu^{7}-2551472\nu^{6}+1399344\nu^{5}+9325040\nu^{4}-6036976\nu^{3}\\ \;-17004560v^{2}+21988752v-7282944)b_{0}^{8}+(-11700v^{7}-575948v^{6}\\ \;+4167164v^{5}-1060860v^{4}-9371740v^{3}+1693660v^{2}+11673108v-6513684)b_{0}^{7}\\ \;+(15080v^{6}+979400v^{5}-4425584v^{4}+382096v^{3}+5710216v^{2}+148136v\\ \;-2809344)b_{0}^{6}+(-7734v^{5}-1038294v^{4}+3202252v^{3}-52244v^{2}-2084822v\\ \;-19158)b_{0}^{5}+(-2064v^{4}+715572v^{3}-1522436v^{2}-9076v+357204)b_{0}^{4}\\ \;+(5851v^{3}-319097v^{2}+451169v-24635)b_{0}^{3}+(-3872\nu^{2}+83624\nu \\ \;-61360)b_{0}^{2}+(1249\nu -9867)b_{0}-170] \end{array},$$

$$\begin{array}{l} b_{10}=\frac{Q^{10}}{2899102924800b_{0}^{14}{\left(\nu b_{0}-b_{0}-1\right)}^{9}}[(22400\nu^{14}+409920\nu^{13}+1014720\nu^{12}-9726080\nu^{11}\\ \;-3521280\nu^{10}+86385600\nu^{9}-111330240\nu^{8}-171037440\nu^{7}+582744960\nu^{6}\\ \;-550034240\nu^{5}+35112000\nu^{4}+348136320v^{3}-304353280v^{2}+112365120v\\ \;-16188480)b_{0}^{14}+(-201600\nu^{13}-3890880\nu^{12}-13023360\nu^{11}+74511360v^{10}\\ \;+106202880\nu^{9}-671267520\nu^{8}+330704640\nu^{7}+1870041600\nu^{6}-3374663040\nu^{5}\\ \;+1575645120\nu^{4}+1259637120\nu^{3}-1873589760\nu^{2}+865589760v-145696320)b_{0}^{13}\\ \;+(877424\nu^{12}+13856448\nu^{11}+97175520\nu^{10}-341615296\nu^{9}-489544432\nu^{8}\\ \;+2072814464\nu^{7}+273949760\nu^{6}-6299616640\nu^{5}+6738156176v^{4}-14196288v^{3}\\ \;-4216050208v^{2}+2722935872v-558742800)b_{0}^{12}+(-1833472\nu^{11}-28624640\nu^{10}\\ \;-377462272\nu^{9}+970592000\nu^{8}+1233044480\nu^{7}-3745627648v^{6}-2152600576v^{5}\\ \;+10025887232\nu^{4}-5730388480\nu^{3}-3346656000v^{2}+4325224960v-1171555584)b_{0}^{11}\\ \;+(2530256\nu^{10}+27659168\nu^{9}+948859856\nu^{8}-1906270080\nu^{7}-2010922592\nu^{6}\\ \;+4592590016\nu^{5}+3483742752v^{4}-8686119296v^{3}+1747844240v^{2}+3209060512v\\ \;-1408974832)b_{0}^{10}+(-2272896\nu^{9}+10991840\nu^{8}-1652145088v^{7}+2664400960\nu^{6}\\ \;+2358232384\nu^{5}-4169356544\nu^{4}-2792458560v^{3}+4314276288v^{2}+146218560v\\ \;-877886944)b_{0}^{9}+(1293024\nu^{8}-83384976v^{7}+2090074736\nu^{6}-2716072144\nu^{5}\\ \;-2029579664\nu^{4}+2826419792\nu^{3}+1222698832v^{2}-1193075440v-118374160)b_{0}^{8}\\ \;+(-412456v^{7}+144241880v^{6}-1941687272v^{5}+2014558744v^{4}+1251889736v^{3}\\ \;-1368033976v^{2}-231963960v+131407304)b_{0}^{7}+(223816v^{6}-151574252v^{5}\\ \;+1326402684v^{4}-1088660824v^{3}-512170784v^{2}+437971268v-12191908)b_{0}^{6}\\ \;+(-430984v^{5}+108223300v^{4}-651169928v^{3}+410820848v^{2}+124004176v\\ \;-68861812)b_{0}^{5}+(514053v^{4}-53453864v^{3}+219557418v^{2}-101677512v-8835455)b_{0}^{4}\\ \;+(-350854v^{3}+17631250v^{2}-45524858v+12106846)b_{0}^{3}+(145077\nu^{2}-3525540\nu \\ \;+4378799)b_{0}^{2}+(-34588v+326224)+3700] \end{array},$$

$$\begin{array}{l} b_{12}=\frac{1}{1376256b_{0}{}^{12}(vb_{0}-b_{0}-1)}\{(-2752512\;v+9830400)b_{6}{}^{2}b_{0}{}^{12}-3584\;Q^{6}b_{2}{}^{3}b_{0}{}^{3}\\ \;+[(-24576\;Q^{2}v^{2}-237568\;Q^{2}v+1179648\;Q^{2})b_{0}{}^{7}+(-18432\;Q^{2}v-239616\;Q^{2})b_{0}{}^{6}\\ \;-6144\;Q^{2}b_{0}{}^{5}]b_{2}{}^{5}+[(-1280\;Q^{4}v^{2}+109824\;Q^{4})b_{0}{}^{6}+8960\;Q^{4}b_{0}{}^{4}]b_{2}{}^{4}+576\;Q^{8}b_{2}{}^{2}b_{0}{}^{2}\\ \;+\{(1280\;Q^{4}v^{2}-5376\;Q^{4})b_{0}{}^{8}+2560\;Q^{4}b_{0}{}^{6}+[(-90112\;Q^{2}v^{2}-696320\;Q^{2}v\\ \;+5308416\;Q^{2})b_{0}{}^{9}+(-55296\;Q^{2}v-718848\;Q^{2})b_{0}{}^{8}-12288\;Q^{2}b_{0}{}^{7}]b_{2}\}b_{4}{}^{2}-40\;Q^{10}b_{2}b_{0}\\ \;+[(-3670016\;v+8912896)b_{2}b_{0}{}^{12}+(40960\;Q^{2}v^{2}+286720\;Q^{2}v-983040\;Q^{2})b_{0}{}^{11}\\ \;+(-18432\;Q^{2}v-239616\;Q^{2})b_{0}{}^{10}-2048\;Q^{2}b_{0}{}^{9}]b_{10}+\{(-5046272\;v+16580608)b_{4}b_{0}{}^{12}\\ \;+(-3584\;Q^{4}v^{2}+118272\;Q^{4})b_{0}{}^{9}-1024\;Q^{4}b_{0}{}^{7}+[(24576\;Q^{2}v^{2}+106496\;Q^{2}v\\ \;-1769472\;Q^{2})b_{0}{}^{10}+(36864\;Q^{2}v+479232\;Q^{2})b_{0}{}^{9}+6144\;Q^{2}b_{0}{}^{8}]b_{2}\}b_{8}\\ \;+\{(-384\;Q^{6}b_{0}{}^{5}+[(-73728\;Q^{2}v^{2}-548864\;Q^{2}v+4227072\;Q^{2})b_{0}{}^{9}+(-55296\;Q^{2}v\\ \;-718848\;Q^{2})b_{0}{}^{8}-12288\;Q^{2}b_{0}{}^{7}]b_{2}{}^{2}+[(57344\;Q^{2}v^{2}+303104\;Q^{2}v-3932160\;Q^{2})b_{0}{}^{10}\\ \;+(36864\;Q^{2}v+479232\;Q^{2})b_{0}{}^{9}+6144\;Q^{2}b_{0}{}^{8}]b_{4}+[(4608\;Q^{4}v^{2}-121344\;Q^{4})b_{0}{}^{8}\\ \;+5120\;Q^{4}b_{0}{}^{6}]b_{2}\}b_{6}+(2688\;Q^{6}b_{2}b_{0}{}^{4}+\{(106496\;Q^{2}v^{2}+942080\;Q^{2}v-5603328\;Q^{2})b_{0}{}^{8}\\ \;+(73728\;Q^{2}v+958464\;Q^{2})b_{0}{}^{7}+20480\;Q^{2}b_{0}{}^{6})b_{2}{}^{3}-128\;Q^{8}b_{0}{}^{3}+[(-1024\;Q^{4}v^{2}\\ \;-101376\;Q^{4})b_{0}{}^{7}-15360\;Q^{4}b_{0}{}^{5}]b_{2}{}^{2}\}b_{4}+Q^{12}\} \end{array},$$

$$\begin{array}{l} b_{14}=\frac{1}{7340032\;b_{0}{}^{14}\left(vb_{0}-b_{0}-1\right)}\{(196608\;Q^{2}v^{2}b_{12}+1572864\;Q^{2}vb_{12}-5505024\;Q^{2}b_{12})b_{0}{}^{13}\\ \;+(-90112\;Q^{2}vb_{12}-1351680\;Q^{2}b_{12})b_{0}{}^{12}-8192\;Q^{2}b_{12}b_{0}{}^{11}+32256\;Q^{6}b_{2}{}^{4}b_{0}{}^{4}\\ \;+[(131072\;Q^{2}v^{2}+1540096\;Q^{2}v-7274496\;Q^{2})b_{0}{}^{8}+(90112\;Q^{2}v+1351680\;Q^{2})b_{0}{}^{7}\\ \;+28672\;Q^{2}b_{0}{}^{6}]b_{2}{}^{6}-7680\;Q^{8}b_{2}{}^{3}b_{0}{}^{3}+[(26624\;Q^{4}v^{2}-2004992\;Q^{4})b_{0}{}^{7}-57344\;Q^{4}b_{0}{}^{5}]b_{2}{}^{5}\\ \;+880\;Q^{10}b_{2}{}^{2}b_{0}{}^{2}+[(-196608\;Q^{2}v^{2}-1835008\;Q^{2}v+14417920\;Q^{2})b_{0}{}^{11}+(-90112\;Q^{2}v\\ \;-1351680\;Q^{2})b_{0}{}^{10}-16384\;Q^{2}b_{0}{}^{9}]b_{4}{}^{3}+[(196608\;Q^{2}v^{2}+1277952\;Q^{2}v-16711680\;Q^{2})b_{0}{}^{12}\\ \;+(90112\;Q^{2}v+1351680\;Q^{2})b_{0}{}^{11}+12288\;Q^{2}b_{0}{}^{10}]b_{6}{}^{2}+\{5376\;Q^{6}b_{0}{}^{6}+[(983040\;Q^{2}v^{2}\\ \;+10125312\;Q^{2}v-65077248\;Q^{2})b_{0}{}^{10}+(540672\;Q^{2}v+8110080\;Q^{2})b_{0}{}^{9}\\ \;+122880\;Q^{2}b_{0}{}^{8}]b_{2}{}^{2}+[(20480\;Q^{4}v^{2}-2502656\;Q^{4})b_{0}{}^{9}-61440\;Q^{4}b_{0}{}^{7}]b_{2}\}b_{4}{}^{2}\\ \;+\{(-28311552\;v+99090432)b_{4}b_{0}{}^{14}+(-18432\;Q^{4}v^{2}+718848\;Q^{4})b_{0}{}^{11}-4096\;Q^{4}b_{0}{}^{9}\\ \;+((131072\;Q^{2}v^{2}+720896\;Q^{2}v-10878976\;Q^{2})b_{0}{}^{12}+(180224\;Q^{2}v+2703360\;Q^{2})b_{0}{}^{11}\\ \;+24576\;Q^{2}b_{0}{}^{10})b_{2}\}b_{10}+[(-32505856\;v+130023424]b_{6}b_{0}{}^{14}-1536\;Q^{6}b_{0}{}^{7}+[(-393216\;Q^{2}v^{2}\\ \;-3342336\;Q^{2}v+27131904\;Q^{2})b_{0}{}^{11}+(-270336\;Q^{2}v-4055040\;Q^{2})b_{0}{}^{10}-49152\;Q^{2}b_{0}{}^{9}]b_{2}{}^{2}\\ \;+[(327680\;Q^{2}v^{2}+2097152\;Q^{2}v-27000832\;Q^{2})b_{0}{}^{12}+(180224\;Q^{2}v+2703360\;Q^{2})b_{0}{}^{11}\\ \;+24576\;Q^{2}b_{0}{}^{10}]b_{4}+[(22528\;Q^{4}v^{2}-649216\;Q^{4})b_{0}{}^{10}+20480\;Q^{4}b_{0}{}^{8}]b_{2}\}b_{8}\\ \;+\{10752\;Q^{6}b_{2}b_{0}{}^{6}-512\;Q^{8}b_{0}{}^{5}+[(589824\;Q^{2}v^{2}+5898240\;Q^{2}v-38535168\;Q^{2})b_{0}{}^{10}\\ \;+(360448\;Q^{2}v+5406720\;Q^{2})b_{0}{}^{9}+81920\;Q^{2}b_{0}{}^{8}]b_{2}{}^{3}+[(4096\;Q^{4}v^{2}-1355776\;Q^{4})b_{0}{}^{9}\\ \;-61440\;Q^{4}b_{0}{}^{7}]b_{2}{}^{2}+\{(6144\;Q^{4}v^{2}+497664\;Q^{4})b_{0}{}^{10}+20480\;Q^{4}b_{0}{}^{8}+[(-1048576\;Q^{2}v^{2}\\ \;-9306112\;Q^{2}v+76808192\;Q^{2})b_{0}{}^{11}+(-540672\;Q^{2}v-8110080\;Q^{2})b_{0}{}^{10}\\ \;-98304\;Q^{2}b_{0}{}^{9}]b_{2}\}b_{4}\}b_{6}+\{-43008\;Q^{6}b_{2}{}^{2}b_{0}{}^{5}+4608\;Q^{8}b_{2}b_{0}{}^{4}+[(-720896\;Q^{2}v^{2}\\ \;-7995392\;Q^{2}v+43515904\;Q^{2})b_{0}{}^{9}+(-450560\;Q^{2}v-6758400\;Q^{2})b_{0}{}^{8}-122880\;Q^{2}b_{0}{}^{7}]b_{2}{}^{4}\\ \;+[(-61440\;Q^{4}v^{2}+5296128\;Q^{4})b_{0}{}^{8}+143360\;Q^{4}b_{0}{}^{6}]b_{2}{}^{3}-160\;Q^{10}b_{0}{}^{3}\}b_{4}\\ \;+[(-19922944\;vb_{12}+49807360\;b_{12})b_{0}{}^{14}-48\;Q^{12}b_{0}]b_{2}+Q^{14}\} \end{array},$$

$$c_{2}=-\frac{Q}{4\;b_{0}},$$

$$c_{4}=\frac{Q^{3}}{512\;b_{0}{}^{4}(vb_{0}-b_{0}-1)}[(2\;v^{2}-4\;v+2\;)b_{0}{}^{2}+(2\;-2\;v)b_{0}+1],$$

$$\begin{array}{l} c_{6}=-\frac{Q^{5}}{147456\;b_{0}{}^{7}{\left(vb_{0}-b_{0}-1\right)}^{3}}[(8\;v^{5}-128\;v^{4}+240\;v^{3}-32\;v^{2}-184\;v+96)\;b_{0}{}^{5}\\ \;+(-24\;v^{4}+360\;v^{3}-360\;v^{2}-264\;v+288\;)b_{0}{}^{4}+(44\;v^{3}-420\;v^{2}+132\;v+244\;)b_{0}{}^{3}\\ \;+(232\;v-42\;v^{2}+2\;)b_{0}{}^{2}+(22\;v-60\;)b_{0}-5] \end{array},$$

$$\begin{array}{l} c_{8}=-\frac{Q^{7}}{75497472\;b_{0}{}^{10}{\left(vb_{0}-b_{0}-1\right)}^{5}}[(3216\;v^{7}-15408\;v^{6}+24912\;v^{5}-6000\;v^{4}-29520\;v^{3}\\ \;+39024\;v^{2}-20112\;v+3888\;)b_{0}{}^{8}+(-16080\;v^{6}+60960\;v^{5}-63600\;v^{4}-33600\;v^{3}\\ \;+114000\;v^{2}-81120\;v+19440\;)b_{0}{}^{7}+(-428\;v^{6}+38288\;v^{5}-108684\;v^{4}+60416\;v^{3}\\ \;+94396\;v^{2}-124176\;v+40188\;)b_{0}{}^{6}+(1336\;v^{5}-53608\;v^{4}+109296\;v^{3}-19600\;v^{2}\\ \;-80936\;v+43512)\;b_{0}{}^{5}+(-2096\;v^{4}+47964\;v^{3}-65748\;v^{2}-4012\;v+23892\;)b_{0}{}^{4}\\ \;+(1948\;v^{3}-27136\;v^{2}+22492\;v+2696)\;b_{0}{}^{3}+(-1117\;v^{2}+9128\;v-3815)\;b_{0}{}^{2}\\ \;+(370\;v-1410\;)b_{0}-55] \end{array},$$

$$\begin{array}{l} c_{10}=\frac{Q^{9}}{60397977600\;b_{0}{}^{13}{\left(vb_{0}-b_{0}-1\right)}^{7}}[(1600\;v^{11}+72480\;v^{10}-960960\;v^{9}+3537120\;v^{8}\\ \;-4771200\;v^{7}-880320\;v^{6}\;+9475200\;v^{5}-9445440\;v^{4}+1308480\;v^{3}+3600800\;v^{2}\\ \;-2431680\;v\;+493920\;)b_{0}{}^{11}+(-11200\;v^{10}-518560\;v^{9}+6208160\;v^{8}-18551680\;v^{7}\\ \;+14846720\;v^{6}+21008960\;v^{5}-45317440\;v^{4}+20800640\;v^{3}+11641280\;v^{2}\\ \;-13564320\;v+3457440)\;b_{0}{}^{10}+(23336\;v^{9}+1969928\;v^{8}-19393216\;v^{7}\;\\ \;+44830592\;v^{6}-13689520\;v^{5}-66084016\;v^{4}+71105792\;v^{3}-1117504\;v^{2}\\ \;-27499192\;v+9853800\;)b_{0}{}^{9}+(-17456\;v^{8}-4713840\;v^{7}+37036272\;v^{6}\\ \;-63503984\;v^{5}-7305840\;v^{4}+89499696\;v^{3}-45688880\;v^{2}-19459152\;v\\ \;+14153184)b_{0}{}^{8}+(-33980\;v^{7}+7727068\;v^{6}-47283244\;v^{5}+57453900\;v^{4}\\ \;+25152780\;v^{3}-62541740\;v^{2}+10101212\;v+9424004)b_{0}{}^{7}\;+(116760\;v^{6}\\ \;-8919720\;v^{5}+41681104\;v^{4}-34006096\;v^{3}-21915336\;v^{2}+23022584\;v\\ \;+20704)b_{0}{}^{6}+(-172946\;v^{5}+7335694\;v^{4}-25480732\;v^{3}+12969284\;v^{2}\\ \;+9123182\;v-3774482)\;b_{0}{}^{5}+(159544\;v^{4}-4242372\;v^{3}+10451396\;v^{2}\\ \;-2963324\;v-1562044\;)b_{0}{}^{4}+(-97851\;v^{3}\;+1657497\;v^{2}-2644289\;v\\ \;+355355)\;b_{0}{}^{3}+(39292\;v^{2}-396104\;v+316660)b_{0}{}^{2}+(-9469\;v+44047)\;b_{0}+1050] \end{array},$$

$$\begin{array}{l} c_{12}=-\frac{1}{12\;b_{0}}(14\;Qc_{2}{}^{10}-80\;Qc_{2}{}^{7}c_{4}+36\;Qc_{2}{}^{5}c_{6}+120\;Qc_{2}{}^{4}c_{4}{}^{2}-16\;Qc_{2}{}^{3}c_{8}\\ \;-72\;Qc_{2}{}^{2}c_{4}c_{6}-32\;Qc_{2}c_{4}{}^{3}+10\;Qc_{2}c_{10}+16\;Qc_{4}c_{8}+9\;Qc_{6}{}^{2}+10\;b_{2}c_{10}+8\;b_{4}c_{8}\\ \;+6\;b_{6}c_{6}+4\;b_{8}c_{4}+2\;b_{10}c_{2}) \end{array},$$

$$\begin{array}{l} c_{14}=\frac{1}{7\;b_{0}}(21\;Qc_{2}{}^{12}-140\;Qc_{2}{}^{9}c_{4}+60\;Qc_{2}{}^{7}c_{6}+280\;Qc_{2}{}^{6}c_{4}{}^{2}-24\;Qc_{2}{}^{5}c_{8}-180\;Qc_{2}{}^{4}c_{4}c_{6}\\ \;-160\;Qc_{2}{}^{3}c_{4}{}^{3}+10\;Qc_{2}{}^{3}c_{10}+48\;Qc_{2}{}^{2}c_{4}c_{8}+27\;Qc_{2}{}^{2}c_{6}{}^{2}+72\;Qc_{2}c_{4}{}^{2}c_{6}+8\;Qc_{4}{}^{4}-6\;Qc_{2}c_{12}\\ \;-10\;Qc_{4}c_{10}-12\;Qc_{6}c_{8}-6\;b_{2}c_{12}-5\;b_{4}c_{10}-4\;b_{6}c_{8}-3\;b_{8}c_{6}-2\;b_{10}c_{4}-b_{12}c_{2}) \end{array}.$$
